# The effectiveness of emotion-oriented approaches on psychological outcomes and cognitive function in older adults: A meta-analysis of randomised controlled trials

**DOI:** 10.7189/jogh.14.04123

**Published:** 2024-06-28

**Authors:** Chiao-Ling Lin, Ruey Chen, Christina Yeni Kustanti, Hsin Chu, Chiu-Kuei Lee, Kondwani Joseph Banda, Chien-Mei Sung, Shu-Fen Niu, Shu-Yen Liu, Kuei-Ru Chou

**Affiliations:** 1 School of Nursing, College of Nursing, Taipei Medical University, Taipei, Taiwan; 2 Department of Nursing, Taipei Medical University-Shuang Ho Hospital, New Taipei, Taiwan; 3 Post-Baccalaureate Program in Nursing, College of Nursing, Taipei Medical University, Taipei, Taiwan; 4 Sekolah Tinggi Ilmu Kesehatan Bethesda Yakkum, Yogyakarta, Indonesia; 5 Institute of Aerospace and Undersea Medicine, School of Medicine, National Defense Medical Center, Taipei, Taiwan; 6 Department of Neurology, Tri-Service General Hospital, National Defense Medical Center, Taipei, Taiwan; 7 Endoscopy Unit, Surgery Department, Kamuzu Central Hospital, Ministry of Health, Malawi; 8 Department of Nursing, Fu Jen Catholic University Hospital, Fu Jen Catholic University, New Taipei, Taiwan; 9 Department of Nursing, Shin Kong Wu Ho-Su Memorial Hospital, Taipei, Taiwan; 10 Department of Nursing, Fu Jen Catholic University, New Taipei, Taiwan; 11 Department of Nursing, Taipei Veterans General Hospital, Taipei, Taiwan; 12 Research Center in Nursing Clinical Practice, Wan Fang Hospital Taipei Medical University, Taipei, Taiwan; 13 Psychiatric Research Center, Taipei Medical University Hospital, Taipei, Taiwan; 14 Research Center for Neuroscience, Taipei Medical University, Taipei, Taiwan

## Abstract

**Background:**

Emotion-oriented approaches have demonstrated effectiveness in the care of the elderly. However, related studies have reported conflicting results. We aimed to explore the pooled effect of emotion-oriented approaches on the psychological outcomes and cognitive function of older adults through a meta-analysis of randomised controlled trials (RCTs).

**Methods:**

We searched eight databases – CINAHL, Cochrane, Embase, Ovid MEDLINE, PsycINFO, PubMed, Scopus, and Web of Science – for RCTs from inception to 11 January 2024. Participants aged 60 years or older who received emotion-oriented approaches as the intervention, and reported outcomes of interest in the studies were included. The primary outcome was psychological outcomes (depression, self-esteem, life satisfaction and loneliness), and the secondary outcome was global cognitive function. The pooled effect size was computed in comprehensive meta-analysis 3.0 software using Hedges’ g (g) with random-effects model. Furthermore, heterogeneity was assessed through Cochrane’s Q and *I^2^* tests. The quality of the included studies was evaluated using the Cochrane Risk of Bias tool. To explore potential sources of heterogeneity, moderator analyses were conducted.

**Results:**

We included 37 RCTs and found that emotion-oriented approaches improve depression (g = −0.82, 95% CI = −1.08, −0.56), self-esteem (g = 0.98, 95% CI = 0.31, 1.64), life satisfaction (g = 0.63, 95% CI = 0.37, 0.88), loneliness (g = −2.22, 95% CI = −3.80, −0.64) and global cognitive function (g = 0.34, 95% CI = 0.19, 0.49) in older adults. We also observed significant follow-up effects on depression (g = −1.40, 95% CI = −2.45, −0.34) and loneliness (g = −3.48, 95% CI = 6.02, −0.94).

**Conclusions:**

Emotion-oriented approaches are promising interventions in improving psychological outcomes and global cognitive function in older adults. Health care workers should receive training to promote and integrate emotion-oriented approaches into routine care of older adults emphasising the importance of collaborative efforts among health care professionals and caregivers to ensure holistic care delivery.

The projection for the global population aged ≥60 years will increase from 1.4 billion to 2.1 billion between 2030 and 2050 [1]. Internationally, the increase in the elderly population from 7 to 14% signifies the pace of societal aging. Compared to other countries, France has undergone a gradual aging process over 115 years, while Sweden took 85 years. In contrast, the USA is projected to gradually age over 73 years, whereas Taiwan is expected to reach a similar level of aging in just 24 years [2]. The aging of the population structure will also exacerbate the burden of the working-age individuals (aged 15–64) and according to the US Central Intelligence Agency [3], there was approximately one older adults (65 years and older) supported by every four working-age individuals in the USA in 2021. However, in Japan, with only two working-age individuals supporting one older adult in 2021, the numbers highlighted the population aging issue and social support pressure the country faces. Furthermore, this demographic shift is accompanied by a simultaneous increase in the number of older adults facing challenges such as cognitive decline, physical impairments, and loneliness [4–6].

According to the research, individuals with lower socioeconomic status (SES) experience accelerated aging and more pronounced declines in cognitive performance, as well as in social function, such as organisational membership, number of close friends, volunteering, and cultural engagement [7]. Conversely, higher SES typically correlates with a better quality of life, wider social networks, and improved access to health care resources. These factors, such as achieving educational, participating in leisure and social activities, contribute to increased cognitive stimulation and the development of cognitive reserve, which may aid in preventing or delaying the onset of cognition function decline [8–10]. Concerning loneliness, personality traits have been linked to loneliness among older adults [11]. A meta-analysis indicated that the typical lonely individual tends to exhibit introverted traits [12]. This inclination toward introversion may result in a preference for solitude, thereby reducing opportunities for social interaction and potentially exacerbating feelings of loneliness [13]. Additionally, another significant concern emerging is the prevalence of psychological disturbances, including anxiety, sadness, depression, and disruptions in social interactions [14,15]. The accumulation of these conditions can have adverse effects on health, diminish the quality of life (QoL), and erode overall well-being.

The bidirectional relationship between caregiver stress and the well-being of older adults underscores their interconnectedness. On one hand, the severity of the care recipient’s disease, behavioural issues, functional status, cognitive function, and dependency can impact caregivers, resulting in stress during the care of older adults [16,17]. On the other hand, caregiver stress, resulting from the physical, emotional, and financial demands of caregiving, significantly influences the well-being of older adults [16,18]. This stress can lead to a decline in the quality of care, an increased risk of neglect or abuse, and mistreatment among older adults, as well as a decrease in the well-being of caregivers over time [17,18]. Consequently, the identification of strategies to promote healthy aging and alleviate caregiver burden is urgently required. As a strategic approach, emotion-oriented approaches play a pivotal role in enhancing emotional well-being, social function, and ultimately the QoL among older adults, while also improving knowledge and caregiving skills [19]. These interventions encompass a variety of approaches, including reminiscence therapy, simulated presence therapy, validation therapy, sensory stimulation, and supportive psychotherapy [20]. Emotion-oriented approaches encourage older adults to express their feelings [21–23]. These interactions with others can provide social support, facilitate self-understanding, enhance self-esteem, achieve internal self-integration, develop a sense of life meaning, and boost self-worth [23,24]. Therefore, emotion-oriented approaches are important and useful non-pharmacological interventions to improve psychological well-being and cognition function in older adults by health care workers.

Existing evidence from a prior systematic review [25] revealed inconsistency and lack of clarity among studies on the effects of emotion-oriented approaches in older adults. As a result, a comprehensive review of current studies to generate more plausible evidence was required because no conclusive evidence on the effects of emotion-oriented approaches on psychological and cognitive outcomes in older adults could be identified. Moreover, previous studies have shown that reminiscence therapy significantly improved depression, self-esteem, life satisfaction, improved cognitive function, quality of life, depressive, and neuropsychiatric symptoms among people with dementia [21,26,27]. Lan et al. [22], and Westerhof and Slatman [28] indicated that life review alleviates depression and improves well-being among older adults with no significant effects on life satisfaction, self-esteem, QoL, or autobiographical memory among older adults. Garland et al. [4] revealed that simulated presence therapy improved challenging behaviours among people with dementia. As such, current studies have primarily focused on single approach using reminiscence therapy, life review, simulated presence therapy, and validation therapy, yielding inconsistent results and no meta-analysis study the effectiveness of emotion-oriented approaches in older adults. In addition to the type of intervention [29], several other factors may serve as moderators of treatment effect, including age, gender [30], type of diagnosis, intervention format [29], intervention duration [31], study setting, and sample size [30]. To address the gap in the current literature, we conducted a meta-analysis of randomised controlled trials to explore the effectiveness of emotion-oriented approaches on the psychological outcomes and cognitive function of older adults.

## METHODS

### Reporting standard

We conducted this meta-analysis following the Preferred Reporting Items for Systematic Reviews and Meta-Analyses (PRISMA) [32] and registered it in the International Prospective Register of Systematic Reviews (PROSPERO: CRD42023445974).

### Data sources and search strategy

A comprehensive search was conducted with the assistance of an experienced medical librarian from inception to 11 January 2024, without restrictions on language and publication year, in eight databases: CINAHL, Cochrane, Embase, Ovid Medline, PsycINFO, PubMed, Scopus, and Web of Science. We conducted a forward citation search to identify articles referenced in specific articles, and a backward citation search was carried out to review the reference lists of previous systematic reviews and meta-analyses. Furthermore, a manual search was conducted in Google Scholar and ProQuest to retrieve additional potentially eligible studies. Two authors conducted the literature search and screening independently. The search terms included a combination of Boolean keywords and relevant keywords from Medical Subject Headings (MeSH), Subject Headings, and Emtrees: (aged OR elder OR elderly OR older adults OR seniors) AND (emotion-oriented care OR emotion-oriented OR reminiscence therapy OR life review OR life history review OR simulated presence therapy OR validation therapy) AND (self-esteem OR depression OR life satisfaction OR loneliness OR cognitive function OR cognition OR global cognitive function) AND (randomised controlled trials OR randomised controlled trials OR randomised OR randomised OR randomisation OR randomisation OR randomly). The specific search terms used across the eight databases are outlined in Table S1 in the **Online Supplementary Document**, and the corresponding syntaxes are presented in Table S2 in the **Online Supplementary Document**. Differences in screening decisions were resolved by consultation with a third investigator. In cases where the required data were not available in the published articles, we contacted the corresponding authors to obtain the original and missing data.

### Eligibility criteria

Eligibility for inclusion in our meta-analysis was determined on the basis of the population, intervention, comparison, outcome, and study design (PICOs) framework: Population: older adults with an age of ≥60 years (without specific restrictions in their condition); Intervention: emotion-oriented approaches (reminiscence therapy, life review, validation therapy, simulated presence therapy or supportive psychotherapy); Comparison: no intervention, usual care, wait-list, or placebo; Outcomes: (1) changes in psychological outcomes: depression, self-esteem, life satisfaction, and loneliness; (2) global cognitive function; Study design: Randomized Controlled Trials (RCTs). The following studies were excluded: (1) case reports or literature reviews; (2) systematic reviews or meta-analyses; (3) book chapters, letters to the editor, abstracts, or study protocols; (4) studies with insufficient data as the authors did not provide the requested information following email inquiries at the time of the analysis.

### Data extraction

Two reviewers (CLL and KJB) independently extracted data from each of the included studies. To maintain the consistency across the reviewer, we used pre-specified guidelines for data extraction including study characteristics (first author, author information, publication year, and country where researchers conducted the study), participants characteristics (sample size, mean age and gender), interventions (type, total week, deliver format, length of session, setting, and comparator information), and outcomes (the relevant statistics at the endpoint of the intervention for estimating effect sizes, such as mean, standard deviation, and measurement tools). We derived the mean and standard deviation from incomplete statistical data by utilising the sample size, median, standard error, interquartile range, and *P*-value, following the guidelines outlined in the Cochrane handbook [33]. When dealing with multiple sets of posttreatment data collected at different time points, we selected the outcome reported at the conclusion of the intervention for statistical analysis. Discrepancies among the reviewers were resolved through discussion with an expert reviewer (KRC).

### Quality assessment of included studies

To assess the methodological quality of the included studies, we employed the Cochrane risk of bias tool for randomised trials version 2 (RoB 2.0) [34], which evaluates each included study based on five key domains: (1) randomisation process, (2) deviation from intended interventions, (3) missing outcome data, (4) measurement of outcome, and (5) selection of the reported results. (1) The randomisation process domain comprises five questions, (2) deviation from intended intervention domain comprises six questions, (3) missing outcome data domain comprises four questions, (4) measurement of outcome domain comprises five questions, and (5) selection of the reported results domain comprises three questions. To assess the risk of bias across the domains, each question has the following five response options including ‘yes,’ ‘probably yes,’ ‘no information,’ ‘probably no,’ and ‘no,’. The risk of bias judgment for each domain were categorised as ‘low risk of bias,’ ‘some concerns,’ or ‘high risk of bias’. The overall risk of bias for each included study was rated as low risk of bias: when all domains were rated as having low risk of bias, some concerns in the risk of bias: if at least one domain was rated as some concerns, and high risk of bias: if one domain was rated as high risk of bias. In the present study, two reviewers (CLL and KJB) independently assessed the included studies and assigned quality scores. Any disagreements or discrepancies between CLL and KJB were thoroughly discussed and resolved through consensus with a third expert reviewer (KRC) with expertise in methodological quality assessment, familiarity with the Cochrane risk of bias tool, and impartiality in resolving discrepancies.

### Data synthesis and analysis

We assessed the validity and suitability of the scales used in each trial. In cases where multiple scoring systems were employed within a single trial, we adopted a consensus-based approach among the authors to select the most appropriate index test for inclusion in our meta-analysis. Quantitative analysis was performed using Comprehensive Meta-Analysis software (CMA) version 3 using post-treatment mean and standard deviation (SD) for the intervention and control groups in each study. If any of the included studies reported their data in form of *P*-value, median, standard error, or interquartile range, we converted them to mean and SD accordingly [33]. After obtaining the means and SDs of each study, the effect size for each study was calculated using Hedges’ g for the different measurement scales and the pooled effect estimate for each outcome was also presented as Hedges’ g with associated 95% confidence interval (CI) using the random-effects model [35]. The Hedge’s g was calculated by dividing the differences in means by the weighted pooled standard deviation [36], with the pooled effect size estimate interpreted as follows: 0.2, 0.5, and 0.8 indicating small, moderate, and large effect sizes, respectively [37]. At least two studies were necessary to pool data for each outcome in quantitative analysis. Effects were assessed based on measurement time points, including post-intervention effects (immediately after the intervention) and follow-up effects (up to eight to 12 weeks after the intervention).

Heterogeneity was assessed using *I^2^*, and Cochrane’s Q-statistics. The *I^2^* statistic represents the percentage of variation across studies attributed to heterogeneity, with values of 25, 50, and 75% indicating low, moderate, and high heterogeneity, respectively. The Cochrane’s Q-statistics identified heterogeneity among the studies by presenting a Q value with statistically significant result (*P* < 0.10) indicating that the true effect size is not the same for all studies [38]. In the present of significant statistical heterogeneity, we conducted moderator analysis using subgroup and meta-regression analyses to investigate the underlying factors contributing to the identified significant moderate and high heterogeneity. Subgroup analysis was performed for categorical variables with at least two studies in each category. The following categorical variables were included in moderator analysis: type of intervention (life review vs. reminiscence vs. validation therapy), type of diagnosis (depression vs dementia vs. normal cognitive function), intervention format (group vs. individual), intervention duration (≤8 vs. >8 weeks), study setting (community vs. institution), sample size (<30 vs. >30). The following continuous variables were included in the meta-regression analysis: age, percentage of female, and length of session (minutes). Statistical significance was defined as a two-tailed *P-*value of <0.05.

Publication bias was assessed through visual examination of funnel plots and the Egger regression test. Funnel plots depict trial size plotted against the reported effect size, with asymmetry indicating potential publication bias. Additionally, a *P*-value exceeding 0.1 of the Egger’s regression results suggested no presence of publication bias. Furthermore, the trim-and-fill method was used to estimate missing studies and adjust their potential impact on the pooled effect size [39]. Publication bias analysis was conducted only for outcomes with more than 10.

To ensure the robustness of our study findings, we conducted sensitivity analysis using the CMA software by automatically removing one study from pooled analyses and exclusion of high risk of bias studies from the analysis. The results were then compared to the initial pooled effect size analysis to assess consistency.

### Ethical approval

We did not require ethical approval, as we utilised secondary data from previously published studies that had obtained informed consent from participants.

## RESULTS

### Study selection

**Figure 1:**
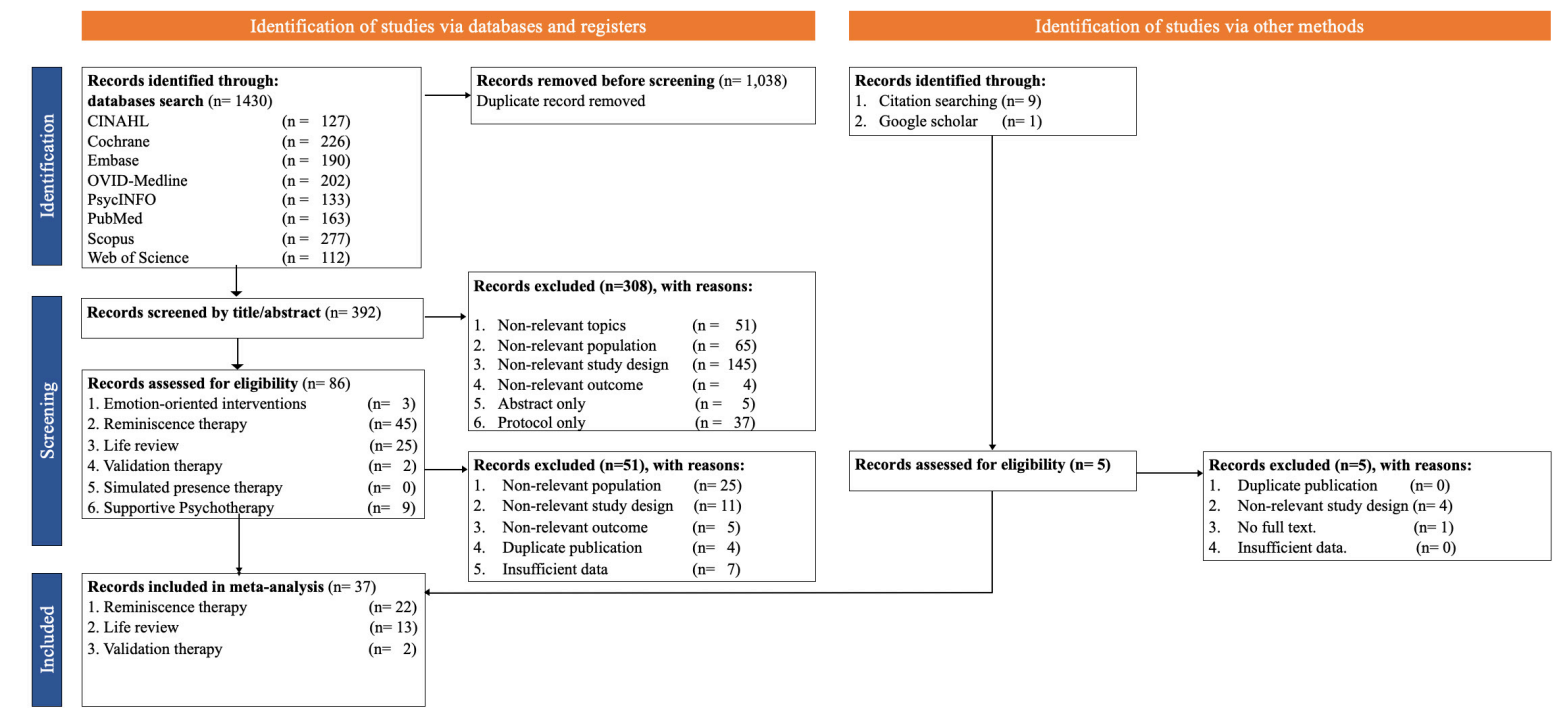
PRISMA flowchart for study selection.

We retrieved 1430 relevant articles from eight databases and located 10 more by searching the references of the retrieved literature. We imported the references into EndNote 20 (Clarivate Analytics, London, UK) and utilised it to eliminate 1038 duplicate articles. After the title and abstract screening based on inclusion and exclusion criteria, 308 articles were excluded. An additional 51 articles were excluded after reviewing the full text. Additionally, one article was found through manual search in Google Scholar, and nine articles were identified through citation searches in previous meta-analyses. However, five articles were excluded due to non-relevant study design. Finally, 37 RCTs were included in the meta-analysis (**Figure 1**, Table S3 in the **Online Supplementary Document**).

### Study characteristics

A total of 2425 older adults were finally included from the randomised controlled trial. Among the 37 randomised controlled trials, 26 analysed depression, seven analysed self-esteem, 13 analysed life satisfaction, four analysed loneliness, and 12 analysed global cognitive function. Most of the studies (n = 22, 64.3%) were conducted in Asian countries, followed by European countries (n = 10, 32.6%) and North America (n = 5, 7.1%). Reminiscence therapy was employed as an intervention in 22 of the included studies, while life review was utilised in 13 studies. Additionally, one studies incorporated validation therapy. Furthermore, one study utilised both reminiscence therapy and validation therapy. However, among older adults, no randomised controlled trials specifically adopted ‘emotion-oriented approaches,’ ‘supportive psychotherapy,’ or ‘simulated presence therapy’ as separate interventions to investigate psychological outcomes and global cognitive function. Most of the trials (n = 27) delivered the interventions in a group format, whereas the remaining 10 studies employed individual formats. The number of intervention sessions ranged from four to 26. These sessions were typically held weekly, with durations ranging from 30 to 120 minutes (**Table 1**, Table S4 in the **Online Supplementary Document**).

**Table 1:** Demographics of included studies

	Number of studies	Number of participants (%)
Sample size	37	2425
Age, mean *±* SD	27	76.69 *±* 6.53
Gender		
*Male*	31	623 (25.7)
*Female*	32	1473 (60.7)
Countries geographic area		
*Asia*	22	1561 (64.3)
*Europe*	10	792 (32.6)
*North America*	5	172 (7.1)
Study setting		
*Community*	17	1320 (54.4)
*Institution*	19	957 (39.5)
*Both*	1	148 (6.1)
Type of diagnosis		
*Dementia*	13	1210 (49.9)
*Depression*	12	569 (23.4)
*Normal cognitive function*	6	336 (13.9)
*Others*	6	310 (12.8)
Type of intervention		
*Life review*	12	261 (21.5)
*Reminiscence*	24	926 (76.2)
*Validation therapy*	2	29 (2.3)
Outcomes		
*Depression*	26	1833 (75.6)
*Self-esteem*	7	773 (31.9)
*Life satisfaction*	13	306 (12.6)
*Loneliness*	4	1090 (44.9)
*Global cognitive function*	12	346 (14.3)

SD – standard deviation

### Risk of bias

In general, 37 studies were included. Steve studies had a low risk of bias (18.9%). The remaining 28 studies were classified as having some concern (75.7%) and two high risk of bias (5.4%), respectively, due to the randomisation process (no detailed information regarding allocation concealment) (Figure S1 in the **Online Supplementary Document****)**.

### Effectiveness of emotion-oriented approaches

#### Depression

**Figure 2:**
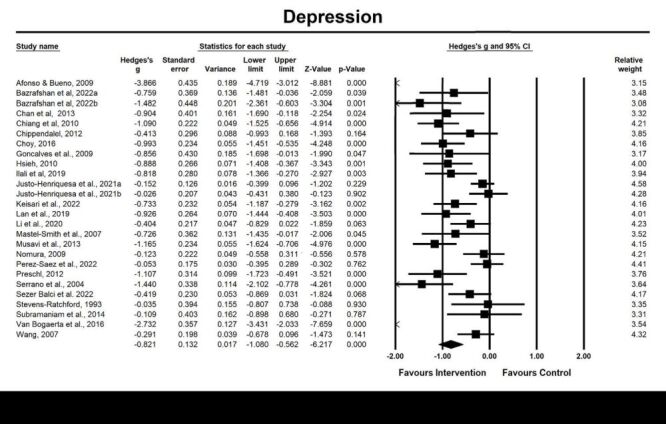
Effect on depression. Test for heterogeneity: Q value = 165.62, df = 25 (*P* < 0.001), *I^2^* = 84.91%. Test for overall effect: Z = −6.22 (*P* < 0.001)

**Figure 3:**
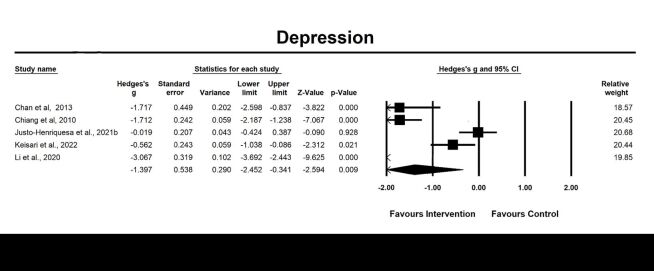
Effect on depression follow-up. Test for heterogeneity: Q value = 78.57, df = 4 (*P* < 0.001), *I^2^* = 94.91%. Test for overall effect: Z = −2.59 (*P* = 0.009).

We included 26 studies for post-intervention analysis and five studies for follow-up analysis on depression (**Figure 2**, **Figure 3**). The results revealed a significant large pooled effect size at post-intervention assessment (g = −0.82, 95% CI = −1.08, −0.56; *P* < 0.001, *I^2^* = 84.90%) and follow-up effects (g = −1.40, 95% CI = −2.45, −0.34; *P* = 0.009, *I^2^* = 94.91%) of emotion-oriented approaches on depression, suggesting that emotion-oriented approaches could alleviate depression and these beneficial effects could be maintained up to eight to 12 weeks after the intervention. Moderator analysis revealed that type of diagnosis (dementia vs. depression vs. normal cognitive function vs. others, *P* < 0.05), was a significant moderator of the post-intervention effect of emotion-oriented approaches on depression (**Table 2****)**.

**Table 2:** The summary of moderator analysis (subgroup analysis)

Outcomes	No. of studies	Hedges’g	95% confidence interval	*P*-value	
				**Each category**	**Between group**
**Type of intervention**					
**Depression**					
Post-intervention effects					0.555
*Life review*	11	−0.760	−0.981, −0.539	<0.001	
*Reminiscence*	15	−0.893	−1.274, −0.511	<0.001	
**Self-esteem**					
Post-intervention effects					0.207
*Life review*	4	0.571	0.074, 1.068	0.024	
*Reminiscence*	3	1.838	−0.065, 3.741	0.058	
**Depression**					
Post-intervention effects					0.555
*Life review*	11	−0.760	−0.981, −0.539	<0.001	
*Reminiscence*	15	−0.893	−1.274, −0.511	<0.001	
Follow-up effects					0.625
*Life review*	2	−1.078	-2.203, 0.048	0.061	
*Reminiscence*	3	−1.586	-3.287, 0.114	0.067	
**Life satisfaction**					
Post-intervention effects					0.585
*Life review*	6	0.569	0.305, 0.832	<0.001	
*Reminiscence*	7	0.707	0.287, 1.126	0.001	
**Cognitive function**					
Post-intervention effects					0.266
*Reminiscence*	10	0.363	0.212, 0.514	<0.001	
*Validation therapy*	2	−0.008	−0.642, 0.627	0.981	
**Type of diagnosis**					
**Depression**					
Post-intervention effects					0.024
*Dementia*	9	−0.599	−1.008, −0.189	0.004	
*Depression*	12	−1.177	−1.579, −0.775	<0.001	
*Normal cognitive function*	3	−0.580	−1.062, −0.099	0.018	
*Others*	2	−1.321	−0.710, 0.067	0.105	
**Self-esteem**					
Post-intervention effects					0.454
*Normal cognitive function*	4	0.644	0.188, 1.101	0.006	
*Others*	2	2.385	−2.151, 6.921	0.303	
**Life satisfaction**					
Post-intervention effects					0.154
*Depression*	5	0.445	0.080, 0.810	0.017	
*Normal cognitive function*	4	1.130	0.434, 1.827	0.001	
*Others*	3	0.394	0.111, 0.677	0.006	
**Intervention format**					
**Depression**					
Post-intervention effects					0.484
*Group*	17	−0.753	−0.920, −0.586	<0.001	
*Individual*	9	−0.990	−1.638, −0.343	0.003	
Follow-up effects					0.384
*Group*	3	−1.768	−3.105, −0.431	0.010	
*Individual*	2	−0.821	−2.483, 0.841	0.333	
**Life satisfaction**					
Post-intervention effects					0.897
*Group*	11	0.641	0.356, 0.926	<0.001	
*Individual*	2	0.588	−0.169, 1.344	0.128	
**Cognitive function**					
Post-intervention effects					0.046
*Group*	7	0.204	0.019, 0.388	0.030	
*Individual*	5	0.471	0.283, 0.660	<0.001	
**Intervention duration**					
**Depression**					
Post-intervention effects					0.461
*≤8 weeks*	15	−0.746	−0.962, −0.530	<0.001	
*>8 weeks*	11	−0.955	−1.477, −0.434	<0.001	
Follow-up effects					0.551
*≤8 weeks*	2	−1.714	−2.132, −1.296	<0.001	
*>8 weeks*	3	−1.200	−2.835, 0.435	0.150	
**Life satisfaction**					
Post-intervention effects					0.585
*≤8 weeks*	11	0.614	0.319, 0.909	<0.001	
*>8 weeks*	2	0.747	0.372, 1.122	<0.001	
**Cognitive function**					
Post-intervention effects					0.685
*≤8 weeks*	5	0.389	0.121, 0.656	0.004	
*>8 weeks*	7	0.321	0.135, 0.508	0.001	
**Study setting**					
**Depression**					
Post-intervention effects					0.723
*Community*	12	−0.916	−1.346, −0.485	<0.001	
*Institution*	13	−0.816	−1.134, −0.505	<0.001	
**Self-esteem**					
Post-intervention effects					0.077
*Community*	4	1.507	0.346, 2.668	0.011	
*Institution*	3	0.408	0.038, 0.778	0.030	
**Life satisfaction**					
Post-intervention effects					0.218
*Community*	6	0.476	0.236, 0.716	<0.001	
*Institution*	7	0.809	0.337, 1.281	0.001	
**Loneliness**					
Post-intervention effects					0.322
*Community*	2	−4.561	−12.522, 3.400	0.261	
*Institution*	2	−0.508	−1.444, 0.429	0.288	
**Cognitive function**					
Post-intervention effects					0.959
*Community*	6	0.353	0.204, 0.502	<0.001	
*Institution*	5	0.339	−0.155, 0.834	0.179	
**Sample size**					
**Depression**					
Post-intervention effects					0.472
<30	6	−0.671	−1.092, −0.249	0.002	
>30	20	−0.860	−1.160, −0.560	<0.001	
**Life satisfaction**					
Post-intervention effects					0.493
*<30*	2	0.783	0.263, 1.303	0.003	
*>30*	10	0.574	0.279, 0.869	<0.001	
**Cognitive function**					
Post-intervention effects					0.616
*<30*	2	0.498	−0.136, 1.132	0.123	
*>30*	9	0.331	0.167, 0.494	0.001	

#### Self-esteem

**Figure 4:**
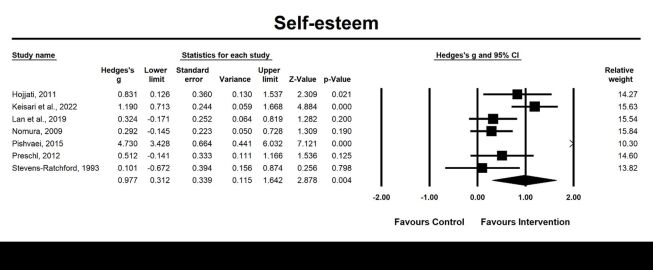
Effect on self-esteem. Test for heterogeneity: Q value = 49.18, df = 6 (*P* < 0.001), *I^2^* = 87.80%. Test for overall effect: Z = 2.87 (*P* = 0.004).

Seven included studies examined self-esteem (**Figure 4**). The results revealed a significant large pooled effect size of 0.98 (95% CI = 0.31, 1.64; *P* = 0.004, *I^2^* = 87.80%) for emotion-oriented approaches on self-esteem, indicating a substantial improvement in self-esteem. Heterogeneity between studies was detected. Moderator analysis revealed that age (*P* < 0.05), and percentage of female (*P* < 0.001) were significant moderators of the post-intervention effect of emotion-oriented approaches on self-esteem (**Table 2**, **Table 3**).

**Table 3:** The summary of moderator analysis (meta-regression)

Outcomes	No. of studies	β coefficient	Standard error	95% confidence interval	*P-*value
**Depression**					
Post-intervention effects					
*Age*	23	0.0324	0.0214	−0.0096, 0.0744	0.1302
*% female*	25	−0.0138	0.0088	−0.0311, 0.0036	0.1191
*Length of session (minutes)*	23	−0.0029	0.0051	−0.0129, 0.0071	0.5692
Follow-up effects					
*Age*	5	−0.0170	0.1294	−0.2706, 0.2367	0.8958
*% female*	4	0.0613	0.0331	−0.0036, 0.1263	0.0643
*Length of session (minutes)*	5	0.0138	0.0300	−0.0451, 0.0726	0.6468
**Self-esteem**					
Post-intervention effects					
*Age*	6	−0.1626	0.0726	−0.3048, −0.0204	0.0250
*% female*	6	−0.0539	0.0146	−0.0825, −0.0254	0.0002
*Length of session (minutes)*	7	−0.0220	0.0181	−0.0574, 0.0134	0.2233
**Life satisfaction**					
Post-intervention effects					
*Age*	11	−0.0312	0.0377	−0.1052, 0.0428	0.4084
*% female*	12	0.0016	0.0082	−0.0145, 0.0178	0.8435
*Length of session (minutes)*	11	−0.0038	0.0085	−0.0205, 0.0128	0.6525
**Cognitive function**					
Post-intervention effects					
*Age*	12	0.0204	0.0152	−0.0095, 0.0502	0.1817
*% female*	10	0.0097	0.0037	0.0023, 0.0170	0.0097
*Length of session (minutes)*	11	−0.0075	0.0125	−0.0321, 0.0171	0.5478

#### Life satisfaction

**Figure 5:**
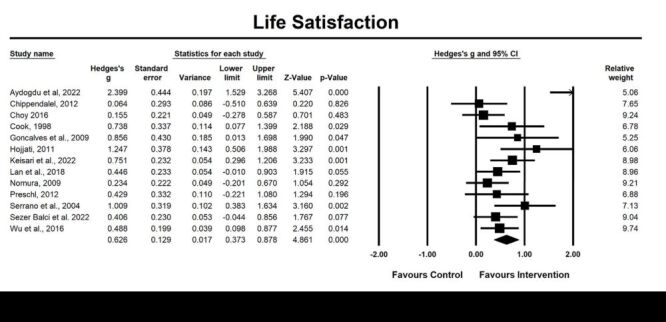
Effect on life satisfaction. Test for heterogeneity: Q value = 33.18, df = 12 (*P* = 0.001), *I^2^* = 63.83%. Test for overall effect: Z = 4.86 (*P* < 0.001).

Thirteen included studies examined life satisfaction. The pooled result of the post-intervention effects for improving life satisfaction was significant (g = 0.63, 95% CI = 0.37, 0.88; *P* < 0.001, *I^2^* = 63.83%) (**Figure 5**), indicating a moderate effect size.

For the moderating effects, type of intervention (life review vs. reminiscence therapy; g = 0.57, *P* < 0.001; g = 0.71, *P* = 0.001), type of diagnosis (depression vs. normal cognitive function vs. others; g = 0.45, *P* = 0.017; g = 1.13, *P* = 0.001; g = 0.39, *P* = 0.006), intervention duration (≤8 vs. >8 weeks; g = 0.61, *P* < 0.001; g = 0.75, *P* = 0.001), and study setting (community vs. institution; g = 0.48, *P* < 0.001; g = 0.81, *P* = 0.001) had significant effects on the improvement of life satisfaction in older adults; however, the difference was not significant (*P* > 0.05).

#### Loneliness

**Figure 6:**
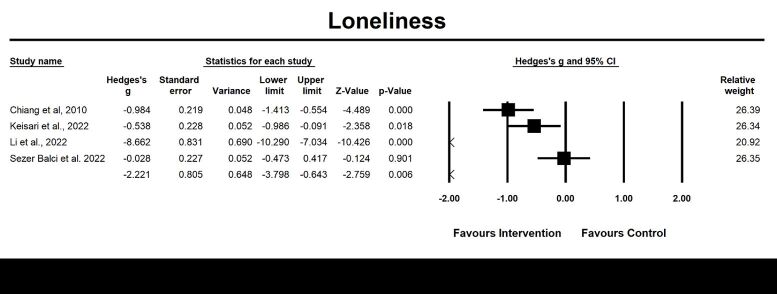
Effect on loneliness. Test for heterogeneity: Q value = 102.74, df = 3 (*P* < 0.001), *I^2^* = 97.08%. Test for overall effects: Z = −2.76 (*P* = 0.006).

**Figure 7:**
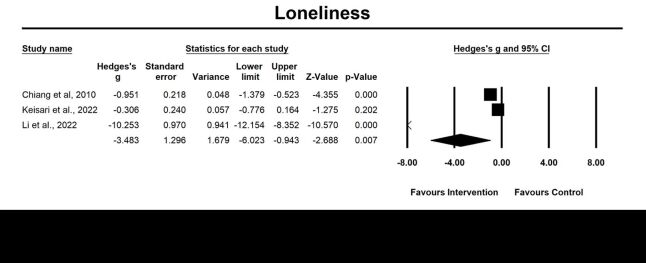
Effect on loneliness follow-up. Test for heterogeneity: Q value = 99.16, df = 2 (*P* < 0.001), *I^2^* = 97.98%. Test for overall effect: Z = 5.80 (*P* < 0.001).

Four randomised controlled trials were evaluated for post-intervention effects, and three were examined for follow-up effects of emotion-oriented approaches on loneliness. The results revealed a significant large pooled effect size at post-intervention assessment (g = −2.22, 95% CI = −3.80, –0.64; *P* = 0.006, *I*^2^ = 97.08%) (**Figure 6**), and follow-up effects (g = −3.48, 95% CI = −6.02, −0.94; *P* = 0.007, *I*^2^ = 97.98%) (**Figure 7**). However, no significant moderator variable was identified for loneliness.

#### Global cognitive function

**Figure 8:**
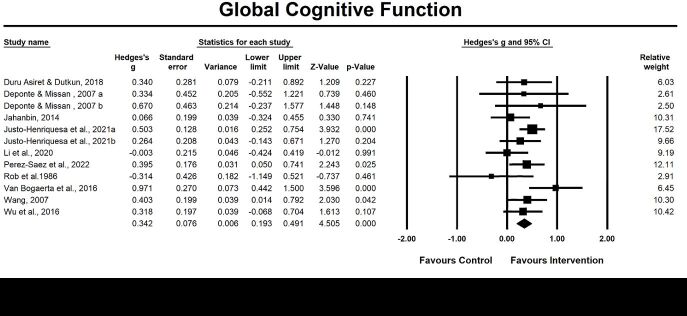
Effect on global cognitive function. Test for heterogeneity: Q value = 14.71, df = 11 (*P* = 0.196), *I^2^* = 25.21. Test for overall effect: Z = 4.51 (*P* < 0.001).

**Figure 9:**
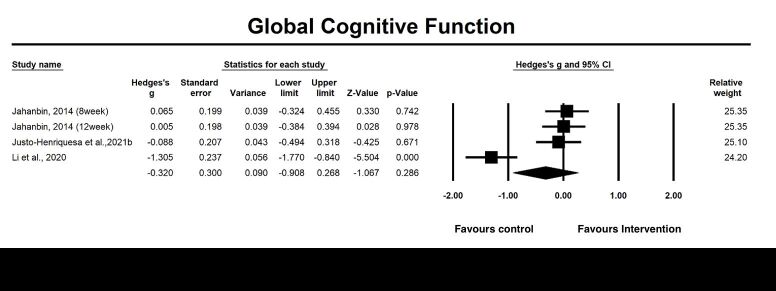
Effect on global cognitive function follow-up. Test for heterogeneity: Q value = 24.59, df = 3 (*P* < 0.001), *I^2^* = 87.80%. Test for overall effect: Z = −1.07 (*P* = 0.286).

Twelve included studies investigated the post-intervention effects, and four studies examined the follow-up effects of emotion-oriented approaches on global cognitive function. The pooled results revealed that emotion-oriented approaches significantly improved global cognitive function at the post-intervention assessment (g = 0.34, 95% CI = 0.19, 0.49; *P* = 0.001, *I*^2^ = 25.21%) (**Figure 8**), indicating a moderate effect size of emotion-oriented approaches on global cognitive function. The results of moderator analysis indicated that heterogeneity resulted from study format (group vs. individual; g = 0.20, *P* = 0.03; g = 0.47, *P* < 0.05), percentage of female (*P* < 0.01) (**Figure 9**). The pooled results of four studies examining the follow-up effects (*P* = 0.286) of emotion-oriented approaches on global cognitive function was not significant.

### Sensitivity analysis

We conducted sensitivity analysis by excluding studies with a high risk of bias and applying a one-study-removed technique. The outcome remained unchanged, indicating that the results of this meta-analysis can be considered relatively robust (Table S5 in the **Online Supplementary Document**).

### Publication bias

For depression, the funnel plot visual assessment appeared to be asymmetrical. The Egger’s regression test yielded a *P*-value of 0.001, indicating the presence of publication bias. To address this, the trim-and-fill test was performed, revealing an adjusted effect size of −1.15 after incorporating eight additional studies, with a 95%CI ranging from −1.46 to −0.84. This adjustment showed no significant difference from the primary value of the pooled effect size, indicating no significant publication bias (Figure S2 and Table S6 in the **Online Supplementary Document**).

Regarding life satisfaction, the funnel plot visual assessment appeared to be asymmetrical. The Egger’s regression test indicated a *P*-value of 0.011, suggesting publication bias. Subsequently, the trim-and-fill test was conducted, revealing an adjusted effect size of 0.41 after incorporating four additional studies, with a 95% CI ranging from 0.12 to 0.69. This adjustment also showed no significant difference from the primary value of the pooled effect size, indicating no significant publication bias (Figure S3 and Table S6 in the **Online Supplementary Document**).

Regarding global cognitive function, the funnel plot visual assessment appeared to be symmetrical. The Egger’s regression test indicated no evidence of bias (*P* = 0.635) (Figure S4 and Table S6 in the **Online Supplementary Document****)**.

## DISCUSSION

This meta-analysis demonstrated the substantial post-intervention (immediately after the intervention) effects, ranging in size from moderate to large, of emotion-oriented approaches on depression, self-esteem, life satisfaction, loneliness, and global cognitive function. The effects of emotion-oriented approaches were sustained for up to eight to 12 weeks after the intervention, particularly the effects on depression and loneliness. Based on these findings, health care providers may consider integrating emotion-oriented approaches into older adults’ care programmes.

### Effect of emotion-oriented approaches on depression

Our study investigated the significance of emotion-oriented approaches on depression among older adults. Despite the presence of publication bias detected through Egger’s regression test, our findings remained robust after adjustment using the trim-and-fill method. This suggests that the observed effect for depression was robust and not substantially impacted by missing studies. With a large effect size (g = −0.82, *P* < 0.001), our findings indicate a clinically significant reduction in depression among older adults following emotion-oriented approaches. Consistent with previous meta-analyses [26,30], which observed moderate to large effect sizes for reminiscence therapy and life review on depression in dementia populations, our study supports the efficacy of these approaches. The mechanism for reducing depression is that the structured process of emotion-oriented approaches not only enables older adults to revisit their experiences and express suppressed emotions but also nurtures a positive sense of self-identity [40]. The findings imply that incorporating emotion-oriented approaches into clinical practice may be beneficial for alleviating depression in older adults. Therefore, our study provides strong evidence to support health care providers in developing effective intervention strategies for this population.

Furthermore, significant effects of emotion-oriented approaches were observed, particularly in the subgroup of older adults with depression, and these effects were sustained up to eight to 12 weeks’ follow-up period. This may be attributed to the nature of emotion-oriented approaches, which emphasise interactions with others to adjust emotions and foster a positive attitude [28]. As social interactions play a crucial role in alleviating depressive symptoms and promoting mental well-being among older adults, we suggest that health care providers consider incorporating emotion-oriented approaches into their clinical practice, particularly for older adults with depression or those at risk of developing depressive symptoms. Additionally, interventions that emphasise social interactions and emotional expression could be integrated into mental health programmes to enhance their effectiveness.

### Effect of emotion-oriented approaches on self-esteem

In this study, significant improvement in self-esteem was observed among older adults. This result is consistent with the findings of Westerhof and Slatman [28]. Participating in emotion-focused interventions enabled older adults to express their feelings openly ensuring that this transformative process facilitates the recognition that each older adult has a meaningful life, embraces both moments of honour and sorrow, leading to introspect on their life experiences. Our research results indicate that the effect of emotion-oriented approaches on self-esteem was lower in older individuals by −0.1626. This effect diminishes with increasing age, aligning with the findings of von Soest et al. [41] who noted that self-esteem peaks around the age of 50–60 and gradually declines thereafter. Additionally, we found that the effect of emotion-oriented approaches on self-esteem was lower by −0.0539 in females. The possible explanation for this study finding is that as women become more concerned with physical appearance and social status as they age and thus, more likely to have lower esteem [42]. However, further investigation is needed to fully understand this relationship. Moreover, the observed gender difference may also have cross-cultural implications [43] suggesting that gender-specific considerations should be integrated when delivering emotion-oriented approaches. Therefore, based on our research findings, we recommend introducing emotion-oriented approaches at an earlier stage of the aging process that are gender-specific.

### Effect of emotion-oriented approaches on life satisfaction

Emotion-oriented approaches significantly enhance life satisfaction in older adults. In addition to addressing publication bias through the trim-and-fill method, our study also provides valuable insights into the clinical significance of the observed effect sizes. With a moderate effect size (g = 0.63, *P* < 0.001), our findings indicate a meaningful improvement in life satisfaction among older adults following emotion-oriented approaches. This aligns with similar findings reported by Tam et al. [27], who also observed a small-effect size for reminiscence-based therapy on life satisfaction in cognitively intact older adults. These results collectively suggest that emotion-oriented approaches have the potential to positively impact the life satisfaction of older adults more broadly, underscoring the importance of integrating such approaches into clinical practice. Thus, our study contributes robust evidence to assist health care providers in designing effective intervention plans for older adults.

While moderator analyses did not achieve statistical significance, our study uncovered additional insights into the mechanisms underlying the benefits of emotion-oriented approaches. Older adults engage in a process of reassessing and reinterpreting their past experiences within the context of their current stage during the intervention. This process fosters a heightened sense of self-control, a profound understanding of the meaning of life, and a reduced sense of dissatisfaction and boredom with their current life circumstances. The mechanisms underpinning these benefits lie in the fact that emotion-oriented approaches promote social interactions, establish a sense of closeness, and stimulate reflection [44]. As a result, they contribute to an overall enhanced sense of meaning and satisfaction in later life [45]. Based on these findings, emotion-oriented approaches could be a promising non-pharmacological method to improve psychological outcomes in older adults.

### Effect of emotion-oriented approaches on loneliness

Our meta-analysis demonstrates that emotion-oriented approaches significantly alleviate loneliness, both in the post-intervention and in follow-up assessments up to 12 weeks after the intervention, among older adults. These results were consistent with that of previous studies, Xu et al. [46] summarised that reminiscence therapy on psychological outcome among older adults demonstrated positive outcomes. This effect is attributed to several factors related to the nature of loneliness in older adults. Due to physical and psychological limitations, older adults often experience varying degrees of social isolation, leading to reduced satisfaction with social relationships and an exacerbated sense of loneliness. Emotion-oriented approaches play a crucial role in addressing this issue by providing opportunities for interaction and interpersonal communication among participants [47]. This social engagement not only connects older adults facing similar challenges but also imparts a profound sense of companionship, effectively countering feelings of solitude and isolation [48]. As older adults engage in meaningful exchanges regarding their life experiences, they tend to become more candid in expressing their emotions, resulting in reduced loneliness [40].

### Effect of emotion-oriented approaches on global cognitive function

Emotion-oriented approaches effectively enhance global cognitive function in older adults in the post-intervention. The structured sessions of the emotion-oriented approaches utilise memories as a conceptual tool to investigate life events, stimulate memory, and foster cognitive discussions [36]. However, the results of our meta-analysis indicated that emotion-oriented approaches had no carry-over effects in the follow-up assessment. This finding is consistent with that of a meta-analysis by Huang et al. [30], who reported that progressive and gradual brain atrophy may be a contributing factor to this. The possible explanation for the non-significant effects of emotion-oriented approaches in the follow-up period is that age-related progressive decline in cognitive function may out way the post-intervention effects. As such, emotion-oriented approaches are needed to be delivered for a longer period to improve global cognitive function.

For subgroup analysis, significant effects were observed in both individual and group formats in enhancing global cognitive function among older adults indicating the efficacy of emotion-oriented approaches across different delivery methods. However, individual format showed to exhibit greater effectiveness in improving cognitive function compared to the group format. These findings align with the prior literature on reminiscence therapy, as outlined in the review conducted by Woods et al. [49]. The possible reason for the larger effect size in the individual format for global cognitive function could be increased accessibility to the intervention, closer contact with participants, and the establishment of stronger therapeutic relationships [50]. For clinical practice, both individual and group formats of emotion-oriented interventions can be valuable in improving cognitive function among older adults. However, clinicians and researchers should consider the potential benefits of individualised interventions, particularly for older adults with specific intervention goals. Furthermore, we found that, the effect of emotion-oriented approaches on global cognitive function was higher in female older adults by 0.0097. The underlying mechanism could be linked to brain structure and functionality [51]. During the aging process, males exhibited a steeper decline in general cognitive functions compared to females. Based on our findings, more studies to explore the impact of emotion-oriented approaches on global cognitive function and examination of the potential differences between genders should be further investigated.

### Strengths and limitations

The present meta-analysis has several strengths. First, it is the first to synthesise the evidence regarding the impact of emotion-oriented approaches on older adults. Second, we conducted an exhaustive and language-inclusive search to identify eligible studies, meticulously adhering to the PRISMA statement guidelines. Third, we proactively registered the study protocol with PROSPERO, thereby enhancing the transparency and traceability of our investigation and ensuring the accuracy and reliability of the study findings. Furthermore, our meta-analysis encompassed a broad spectrum of psychological outcomes, including depression, self-esteem, life satisfaction, loneliness, and cognitive function. In addition, considering the paucity of evidence regarding the impact of the follow-up effects of emotion-oriented approaches on older adults, our meta-analysis provides a comprehensive understanding of the effectiveness of such interventions in this context.

However, the present study acknowledges some limitations. First, moderate to high heterogeneity existed in several outcomes, influencing result interpretation. Despite this, subgroup and meta-regression analyses were conducted to identify potential sources of heterogeneity. Additionally, the predominantly older adult focus in the included studies may limit applicability to other adult populations. Further research targeting healthy older adults or different age groups could offer valuable insights. Second, some concerns and high risk of bias were observed in some of the included studies. The impact of bias in individual studies on meta-analysis results is significant. If high-risk studies are heavily weighted, they may affect the pooled effect estimate, potentially undermining reliability and validity [52,53]. Including studies with methodological flaws or biases without proper consideration can compromise the strength of evidence, leading to inaccurate conclusions [54]. Therefore, careful assessment of bias risk in individual studies is crucial. Strategies, such as sensitivity analyses, should be considered to mitigate bias impact [55]. In our study, after performing sensitivity by excluding studies with a high risk of bias and applying a one-study-removed technique. The result did not significantly affect the overall outcome. Lastly, regarding follow-up effects, due to the existing literature covering follow-up periods from eight weeks to 12 weeks’ post-intervention, a definitive cut-off point for sustainability of mid-term or long-term effects is yet to be established. Continued research into mid- and long-term effects can offer valuable insights, serving as a reference for health care professionals striving to provide effective and tailored care for older adults.

## CONCLUSIONS

Our meta-analysis shows significant positive effects of emotion-oriented approaches, including reducing depression, enhancing self-esteem, and life satisfaction, alleviating loneliness and improving cognitive function in adults with depression, dementia, and normal cognitive status. To ensure successful integration into clinical practice, health care workers should receive comprehensive education and training in emotion-oriented approaches. Collaborative efforts among health care professionals, including nurses, therapists, and caregivers, are crucial. Regular assessments can customise care plans for older adults, ensuring a holistic and interdisciplinary approach to their well-being.

### Implication for practice

This meta-analysis provides evidence supporting the beneficial effects of emotion-oriented approaches on the psychological and global cognitive function of older adults. Healthcare providers should integrate emotion-oriented approaches into care programmes for older adults, particularly those with depression. The specific type, duration, format, session length, and setting of emotion-oriented approaches may be adapted based on available resources and clinician preferences. Additionally, the success of these approaches is contingent upon the intervention setting, whether in a community or institutional context.

In community settings, emotion-oriented approaches can be customised and tailored to meet individual needs due to their flexibility [48] as they focus on enhancing social support and fostering a sense of belonging [49]. However, challenges such as geographical barriers may hinder implementation efforts in the community [50]. Conversely, institutional settings provide structured environments with professional staff, facilitating the integration of these approaches into care routines and addressing the needs of residents in nursing homes [19]. Nevertheless, institutional routines may restrict flexibility [51]. Understanding the distinct characteristics and challenges of each setting is important for effectively implementing and optimising the benefits of emotion-oriented approaches. Specific recommendations for clinicians and caregivers include: 1. Assessment of older adults’ needs and backgrounds; 2. Implementing individualised care plans that integrate emotion-oriented approaches; 3. Providing education and training on principles and techniques of emotion-oriented approaches [19,56].

Training and resources may include information materials on topics such as empathy and communication skills [57]. Following appropriate training, clinical health care providers, psychologists, and other mental health psychotherapists can integrate evidence-based emotion-oriented approaches into their daily practices, especially in nursing homes and other long-term care facilities. In addition, family caregivers and volunteers can undergo training to deliver these interventions to older adults living in the community. Ongoing training and support for health care providers and caregivers are essential for the successful implementation of emotion-oriented approaches.

## Additional material


Online Supplementary Document

## References

[1] World Health Organization. Ageing and health. 2022. Available: https://www.who.int/news-room/fact-sheets/detail/ageing-and-health. Accessed: 19 March.

[2] Kinsella KG. Older workers, retirement, and pensions: A comparative international chartbook: US Department of Commerce, Economics and Statistics Administration, Bureau; 1995.

[3] Central Intelligence Agency. Dependency ratios. 2021. Available: https://www.cia.gov/the-world-factbook/field/dependency-ratios/. Accessed: 9 April.

[4] Levy SS Thralls KJ Goble DJ Krippes TB Effects of a community-based exercise program on older adults’ physical function, activities of daily living, and exercise self-efficacy: Feeling fit club. J Appl Gerontol. 2020;39:40–9. 10.1177/0733464818760237 29504440

[5] Fitzpatrick JM Tzouvara V Facilitators and inhibitors of transition for older people who have relocated to a long-term care facility: A systematic review. Health Soc Care Community. 2019;27:e57–81. 10.1111/hsc.12647 30239055

[6] Tiilikainen E Seppänen M Lost and unfulfilled relationships behind emotional loneliness in old age. Ageing Soc. 2017;37:1068–88. 10.1017/S0144686X16000040

[7] Steptoe A Zaninotto P Lower socioeconomic status and the acceleration of aging: An outcome-wide analysis. Proc Natl Acad Sci U S A. 2020;117:14911–7. 10.1073/pnas.1915741117 32541023 PMC7334539

[8] Shi L Tao L Chen N Liang H Relationship between socioeconomic status and cognitive ability among Chinese older adults: the moderating role of social support. Int J Equity Health. 2023;22:70. 10.1186/s12939-023-01887-6 37095501 PMC10124054

[9] Deng Q Liu W Inequalities in cognitive impairment among older adults in China and the associated social determinants: a decomposition approach. Int J Equity Health. 2021;20:82. 10.1186/s12939-021-01422-5 33741012 PMC7980641

[10] Stern Y How Can Cognitive Reserve Promote Cognitive and Neurobehavioral Health? Arch Clin Neuropsychol. 2021;36:1291–5. 10.1093/arclin/acab049 34651645 PMC8517622

[11] Tapia-Munoz T Ajnakina O Fancourt D Steptoe A Personality traits and loneliness among older people in the UK: Cross-sectional and longitudinal analysis from the English Longitudinal Study of Ageing. Eur J Pers. 2023;08902070231206196. 10.1177/08902070231206196

[12] Buecker S Maes M Denissen JJA Luhmann M Loneliness and the Big Five Personality Traits: A Meta–Analysis. Eur J Pers. 2020;34:8–28. 10.1002/per.2229

[13] Bull DA Personal Characteristics and Loneliness: Is there a Relationship? International Journal of Social Science and Humanities Research. 2023;11:290–301.

[14] Lee J Sung J Choi M The factors associated with subjective cognitive decline and cognitive function among older adults. J Adv Nurs. 2020;76:555–65. 10.1111/jan.14261 31713894

[15] Wrzus C Hänel M Wagner J Neyer FJ Social network changes and life events across the life span: a meta-analysis. Psychol Bull. 2013;139:53–80. 10.1037/a0028601 22642230

[16] Wang S Cheung DSK Leung AYM Davidson PM Factors associated with caregiving appraisal of informal caregivers: A systematic review. J Clin Nurs. 2020;29:3201–21. 10.1111/jocn.15394 32620034

[17] Hawkley L Zheng B Hedberg EC Huisingh-Scheetz M Waite L Cognitive limitations in older adults receiving care reduces well-being among spouse caregivers. Psychol Aging. 2020;35:28–40. 10.1037/pag0000406 31985247 PMC6989024

[18] Schulz R Beach SR Czaja SJ Martire LM Monin JK Family Caregiving for Older Adults. Annu Rev Psychol. 2020;71:635–59. 10.1146/annurev-psych-010419-050754 31905111 PMC7291827

[19] Finnema E Dröes RM Ettema T Ooms M Adèr H Ribbe M The effect of integrated emotion-oriented care versus usual care on elderly persons with dementia in the nursing home and on nursing assistants: a randomized clinical trial. International Journal of Geriatric Psychiatry. 2005;20:330–43. 10.1002/gps.1286 15799079

[20] Rabins PV Rovner BW Rummans T Schneider LS Tariot PN Guideline Watch (October 2014): Practice Guideline for the Treatment of Patients With Alzheimer’s Disease and Other Dementias. Focus Am Psychiatr Publ. 2017;15:110–28. 10.1176/appi.focus.15106 31997970 PMC6519627

[21] Song D Shen Q Xu T-Z Sun Q-H Effects of group reminiscence on elderly depression: A meta-analysis. Int J Nurs Sci. 2014;1:416–22. 10.1016/j.ijnss.2014.10.001

[22] Lan X Xiao H Chen Y Effects of life review interventions on psychosocial outcomes among older adults: A systematic review and meta-analysis. Geriatr Gerontol Int. 2017;17:1344–57. 10.1111/ggi.12947 28124828

[23] Vasse E Vernooij-Dassen M Spijker A Rikkert MO Koopmans R A systematic review of communication strategies for people with dementia in residential and nursing homes. Int Psychogeriatr. 2010;22:189–200. 10.1017/S1041610209990615 19638257

[24] Ingersoll-Dayton B Kropf N Campbell R Parker M A systematic review of dyadic approaches to reminiscence and life review among older adults. Aging Ment Health. 2019;23:1074–85. 10.1080/13607863.2018.1555696 30596457

[25] Finnema E Dröes RM Ribbe M Van Tilburg W The effects of emotion-oriented approaches in the care for persons suffering from dementia: a review of the literature. Int J Geriatr Psychiatry. 2000;15:141–61. 10.1002/(SICI)1099-1166(200002)15:2<141::AID-GPS92>3.0.CO;2-5 10679846

[26] Saragih ID Tonapa SI Yao CT Saragih IS Lee BO Effects of reminiscence therapy in people with dementia: A systematic review and meta-analysis. J Psychiatr Ment Health Nurs. 2022;29:883–903. 10.1111/jpm.12830 35348260

[27] Tam W Poon SN Mahendran R Kua EH Wu XV The effectiveness of reminiscence-based intervention on improving psychological well-being in cognitively intact older adults: A systematic review and meta-analysis. Int J Nurs Stud. 2021;114:103847. 10.1016/j.ijnurstu.2020.103847 33352435

[28] Westerhof GJ Slatman S In search of the best evidence for life review therapy to reduce depressive symptoms in older adults: A meta-analysis of randomized controlled trials. Clin Psychol (New York). 2019;26:e12301. 10.1111/cpsp.12301

[29] Bohlmeijer E Roemer M Cuijpers Phd P Smit F The effects of reminiscence on psychological well-being in older adults: A meta-analysis. Aging Ment Health. 2007;11:291–300. 10.1080/13607860600963547 17558580

[30] Huang H-C Chen Y-T Chen P-Y Hu SH-L Liu F Kuo Y-L Reminiscence therapy improves cognitive functions and reduces depressive symptoms in elderly people with dementia: a meta-analysis of randomized controlled trials. J Am Med Dir Assoc. 2015;16:1087–94. 10.1016/j.jamda.2015.07.010 26341034

[31] Al-Ghafri BR Al-Mahrezi A Chan MF Effectiveness of life review on depression among elderly: a systematic review and meta-analysis. Pan Afr Med J. 2021;40:168. 10.11604/pamj.2021.40.168.30040 34970410 PMC8683455

[32] Page MJ McKenzie JE Bossuyt PM Boutron I Hoffmann TC Mulrow CD The PRISMA 2020 statement: an updated guideline for reporting systematic reviews. Int J Surg. 2021;88:105906. 10.1016/j.ijsu.2021.105906 33789826

[33] Higgins JPTTJ, Chandler J, Cumpston M, Li T, Page MJ. Welch VA (editors). Cochrane Handbook for Systematic Reviews of Interventions version 6.4. 2023. Available: www.training.cochrane.org/handbook. Accessed: 26 March 2024.

[34] Sterne JA Savović J Page MJ Elbers RG Blencowe NS Boutron I RoB 2: a revised tool for assessing risk of bias in randomised trials. BMJ. 2019;366:l4898. 10.1136/bmj.l4898 31462531

[35] Borenstein M Hedges LV Higgins JP Rothstein HR A basic introduction to fixed-effect and random-effects models for meta-analysis. Res Synth Methods. 2010;1:97–111. 10.1002/jrsm.12 26061376

[36] Brüggemann P Rajguru K Comprehensive Meta-Analysis (CMA) 3.0: a software review. Journal of Marketing Analytics. 2022;10:425–9. 10.1057/s41270-022-00184-5

[37] Cohen J. Statistical power analysis for the behavioral sciences. New York: NY Routledge; 2013.

[38] Higgins JPT Thompson SG Deeks JJ Altman DG Measuring inconsistency in meta-analyses. BMJ. 2003;327:557–60. 10.1136/bmj.327.7414.557 12958120 PMC192859

[39] Duval S Tweedie R Trim and fill: a simple funnel-plot–based method of testing and adjusting for publication bias in meta-analysis. Biometrics. 2000;56:455–63. 10.1111/j.0006-341X.2000.00455.x 10877304

[40] Chao S-Y Liu H-Y Wu C-Y Jin S-F Chu T-L Huang T-S The effects of group reminiscence therapy on depression, self esteem, and life satisfaction of elderly nursing home residents. J Nurs Res. 2006;14:36–45. 10.1097/01.JNR.0000387560.03823.c7 16547904

[41] von Soest T Wagner J Hansen T Gerstorf D Self-esteem across the second half of life: The role of socioeconomic status, physical health, social relationships, and personality factors. J Pers Soc Psychol. 2018;114:945–58. 10.1037/pspp0000123 28150978

[42] Sahlstein E Allen M Sex differences in self-esteem: A meta-analytic assessment. Interpersonal communication research: Advances through meta-analysis. 2002:59-72.

[43] Casale S. Gender Differences in Self-esteem and Self-confidence. The Wiley Encyclopedia of Personality and Individual Differences. Wiley Online Library; 2020. p. 185-9.

[44] Wu Y Xu H Sui X Zeng T Leng X Li Y Effects of group reminiscence interventions on depressive symptoms and life satisfaction in older adults with intact cognition and mild cognitive impairment: A systematic review. Arch Gerontol Geriatr. 2023;114:105103. 10.1016/j.archger.2023.105103 37354738

[45] Bazrafshan M-R Jokar M Soufi O Delam H The effect of structured group reminiscence on depression and anxiety of the elderly female hookah users. J Subst Use. 2022;27:528–34. 10.1080/14659891.2021.1967479

[46] Xu L Li S Yan R Ni Y Wang Y Li Y Effects of reminiscence therapy on psychological outcome among older adults without obvious cognitive impairment: A systematic review and meta-analysis. Front Psychiatry. 2023;14:1139700. 10.3389/fpsyt.2023.1139700 37065888 PMC10098219

[47] Gaggioli A Scaratti C Morganti L Stramba-Badiale M Agostoni M Spatola CA Effectiveness of group reminiscence for improving wellbeing of institutionalized elderly adults: study protocol for a randomized controlled trial. Trials. 2014;15:408. 10.1186/1745-6215-15-408 25344703 PMC4216871

[48] Allen AP Doyle C Roche RA The impact of reminiscence on autobiographical memory, cognition and psychological well-being in healthy older adults. Eur J Psychol. 2020;16:317–30. 10.5964/ejop.v16i2.2097 33680185 PMC7913011

[49] Woods B O’Philbin L Farrell EM Spector AE Orrell M Reminiscence therapy for dementia. Cochrane Database Syst Rev. 2018;3:CD001120. 29493789 10.1002/14651858.CD001120.pub3 PMC6494367

[50] Justo-Henriques SI Marques-Castro AE Otero P Vazquez FL Torres AJ Long-term individual cognitive stimulation program in patients with mild neurocognitive disorder: a pilot study. Rev Neurol. 2019;68:281–9. 30906977 10.33588/rn.6807.2018321

[51] Jockwitz C Wiersch L Stumme J Caspers S Cognitive profiles in older males and females. Sci Rep. 2021;11:6524. 10.1038/s41598-021-84134-8 33753754 PMC7985508

[52] Phillips MR Kaiser P Thabane L Bhandari M Chaudhary V Wykoff CC Risk of bias: why measure it, and how? Eye (Lond). 2022;36:346–8. 10.1038/s41433-021-01759-9 34594009 PMC8807607

[53] Drucker AM Fleming P Chan A-W Research Techniques Made Simple: Assessing Risk of Bias in Systematic Reviews. J Invest Dermatol. 2016;136:e109–14. 10.1016/j.jid.2016.08.021 27772550

[54] Boutron I, Page MJ, Higgins JP, Altman DG, Lundh A, Hróbjartsson A, et al. Considering bias and conflicts of interest among the included studies. In Cochrane Handbook for Systematic Reviews of Interventions (second edition), Higgins JPT, Thomas J, Chandler J, Cumpston M, Li T, Page MJ, Welch VA, editors. Wiley-Blackwell; 2019. p. 177-204.

[55] Marušić MF Fidahić M Cepeha CM Farcaș LG Tseke A Puljak L Methodological tools and sensitivity analysis for assessing quality or risk of bias used in systematic reviews published in the high-impact anesthesiology journals. BMC Med Res Methodol. 2020;20:121. 10.1186/s12874-020-00966-4 32423382 PMC7236513

[56] Burnside I Haight B Reminiscence and life review: Therapeutic interventions for older people. Nurse Pract. 1994;19:55–61. 10.1097/00006205-199404000-00011 8035962

[57] Feil N Validation therapy. Geriatr Nurs. 1992;13:129–33. 10.1016/S0197-4572(07)81021-4 1319934

