# Supplementary material for: The effectiveness of emotion-oriented approaches on psychological outcomes and cognitive function in older adults: A meta-analysis of randomised controlled trials
Source: J Glob Health. 2024 Jun 28;14:04123. doi: 10.7189/jogh.14.04123 (PMC11211973; doi:10.7189/jogh.14.04123)
Supplement: Online Supplementary Document [file jogh-14-04123-s001.pdf]

Supporting information

Supplementary list	Page Number
**Table S1.** Search Terms.	1
**Table S2.** Syntaxes.	5
**Table S3.** List of the included studies.	14
**Table S4.** Characteristics of the Included Studies.	17
**Figure S1.** Risk of bias assessment.	29
**Table S5.** Sensitivity analyses.	30
**Figure S2.** Funnel plot on depression.	31
**Figure S3.** Funnel plot on life satisfaction.	32
**Figure S4.** Funnel plot on global cognitive function.	33
**Table S6.** Publication bias.	34

Table S1. Search Terms.

Databases	Population	Intervention	Comparison	Outcome
**CINAHL**	- aged<br>- elderly [a]<br>- "older adults [a]"	- "emotion-oriented [a]"<br>- "emotion-oriented care [a]"<br>- "Reminiscence Therapy"<br>- "Life History Review"<br>- "Life Review [a]"<br>- "Simulated Presence Therapy [a] "<br>- "Validation Therapy"<br>- "Supportive Psychotherapy [a]"	- no intervention<br>- usual care<br>- wait-list	- "self-esteem [a]"<br>- depression<br>- "life satisfaction [a]"<br>- Loneliness<br>- "cognitive function [a]"<br>- Cognition
**Cochrane**	- aged<br>- elderly<br>- "older adults"	- "Reminiscence Therapy"<br>- "life review"<br>- "emotion-oriented care"<br>- emotion-oriented<br>- "validation therapy"<br>- "simulated presence therapy"<br>- "Supportive psychotherapy"	- no intervention<br>- usual care<br>- wait-list	- "self-esteem"<br>- Depression<br>- "life satisfaction"<br>- loneliness<br>- "cognitive function"<br>- Cognition
**Embase**	- older adults<br>- aged	- "emotion-oriented "	- no intervention<br>- usual care	- "self-esteem"<br>- depression

Databases	Population	Intervention	Comparison	Outcome
	- elderly	- "emotion-oriented care [a]"<br>- "reminiscence therapy"<br>- "life review therapy"<br>- "life review [a]"<br>- "simulated presence therapy"<br>- "validation therapy"<br>- "supportive psychotherapy"	- wait-list	- "life satisfaction"<br>- loneliness<br>- "cognitive function"<br>- Cognition
**OVID-Medline**	- Aged<br>- Elderly<br>- "older adults"	- "emotion oriented"<br>- "emotion-oriented care"<br>- "reminiscence therapy [a]"<br>- "life review"<br>- "simulated presence therapy"<br>- "validation therapy"<br>- "supportive psychotherapy"	- no intervention<br>- usual care<br>- wait-list	- "self-esteem"<br>- depression<br>- "Life satisfaction"<br>- Loneliness<br>- Cognition<br>- "Cognitive function"
**PsycINFO**	- aged<br>- elderly [a]<br>- older adults	- "emotion-oriented [a]"<br>- "emotion-oriented care [a]"<br>- "Reminiscence Therapy"<br>- "life review therapy"<br>- "Life Review [a]"	- no intervention<br>- usual care<br>- wait-list	- "self-esteem [a]"<br>- depression<br>- "life satisfaction [a]"<br>- Loneliness<br>- "Cognitive function [a]"<br>- Cognition

Databases	Population	Intervention	Comparison	Outcome
**PubMed**	- aged<br>- “older adults”<br>- Elderly	- “Simulated Presence Therapy ”<br>- “Validation Therapy”<br>- “Supportive Psychotherapy”<br>- “emotion-oriented care”<br>- “emotion-oriented ”<br>- “Reminiscence Therapy ”<br>- “life review ”	- no intervention<br>- usual care<br>- wait-list	- “self-esteem”<br>- depression<br>- “life satisfaction”<br>- Loneliness<br>- “cognitive function”<br>- cognition
**Scopus**	- “older adults”<br>- Elderly<br>- Aged	- “Simulated Presence Therapy”<br>- “Validation Therapy”<br>- “Supportive Psychotherapy”<br>- “emotion-oriented care”<br>- “emotion-oriented”<br>- “reminiscence therapy”<br>- “life review”<br>- “simulated presence therapy”<br>- “validation therapy”<br>- “Supportive psychotherapy”	- no intervention<br>- usual care<br>- wait-list	- “self-esteem”<br>- Depression<br>- “life satisfaction”<br>- Loneliness<br>- “cognitive function”<br>- Cognition [a]

Databases	Population	Intervention	Comparison	Outcome
**Web of Science**	- "older adults"<br>- Aged<br>- Elderly	- "emotion oriented"<br>- "emotion-oriented care"<br>- "reminiscence therapy"<br>- "life review"<br>- "simulated presence therapy"<br>- "validation therapy"<br>- "supportive psychotherapy"	- no intervention<br>- usual care<br>- wait-list	- "self-esteem"<br>- Depression<br>- "life satisfaction"<br>- loneliness<br>- "cognitive function"<br>- Cognition

[a] *search as keywords*

Table S2. Syntaxes.

Databases	Syntaxes	Number	Total
**CINAHL**	S1: MH aged OR TI aged OR AB aged OR TI elderly OR AB elderly OR TI "older adult" OR AB "older adult"	1,123,885	127
	S2: TI "emotion-oriented" OR AB "emotion-oriented" OR TI "emotion-oriented care" OR AB "emotion-oriented care" OR MH "Reminiscence Therapy" OR "Reminiscence Therapy" OR "Reminiscence Therapy" OR MH “Life History Review” OR TI “Life History Review” OR AB “Life History Review” OR TI “Life Review” OR AB “Life Review”	2,676	
	S3: TI "Simulated Presence Therapy" OR AB "Simulated Presence Therapy" OR MH “Validation Therapy” OR TI “Validation Therapy” OR AB “Validation Therapy” OR TI "Supportive Psychotherapy" OR AB "Supportive Psychotherapy"	287	
	S4: S2 OR S3	2,929	
	S5: TI self-esteem OR AB self-esteem OR MH depression OR TI depression OR AB depression OR TI "life satisfaction" OR AB "life satisfaction" OR MH Loneliness OR TI Loneliness OR AB Loneliness	225,033	
	S6: MH Cognition OR TI Cognition OR AB Cognition OR TI "cognitive function" OR AB "cognitive function"	97,230	
	S7:S5 OR S6	311,020	
	S8: MH Randomized Controlled Trials OR TI Randomized Controlled Trials OR AB Randomized Controlled Trials OR TI Randomised Controlled Trials OR AB	360,370	

Databases	Syntaxes	Number	Total
	Randomised Controlled Trials OR TI Randomized OR AB Randomized OR TI Randomised OR AB Randomised OR TI Randomization OR AB Randomization		
	S9: TI Randomisation OR AB Randomisation OR TI randomly OR AB randomly	126,518	
	S10: S8 OR S9	421,125	
	S11: S1AND S4 and S7 AND S10	127	
**Cochrane**	#1: MeSH descriptor: [Aged] in all MeSH products	256,396	226
	#2: (Elderly OR Aged OR "older adults"):ti,ab,kw	639,979	
	#3: #1 OR #2	640,590	
	#4: ("emotion-oriented care" OR emotion-oriented OR "Reminiscence Therapy" OR "life review" OR "simulated presence therapy" OR "validation therapy" OR "Supportive Psychotherapy"):ti,ab,kw	840	
	#5: MeSH descriptor: [Depression] explode all trees	18,859	
	#6: MeSH descriptor: [Loneliness] explode all trees	258	
	#7: MeSH descriptor: [Cognition] explode all trees	16,657	
	#8: ("self-esteem" OR depression OR "life satisfaction" OR loneliness OR "cognitive function" OR cognition):ti,ab,kw	138,113	
	#9: #5 OR #6 OR #7 OR #8	141,817	
	#10: MeSH descriptor: [Randomized Controlled Trial] explode all trees	25,729	
	#11: MeSH descriptor: [Random Allocation] explode all trees	23,370	

Databases	Syntaxes	Number	Total
	#12: ("Randomized Controlled Trial" OR "Randomised Controlled Trials" OR Randomized OR Randomised OR Randomization OR Randomisation OR randomly):ti,ab,kw	1,251,317	
	#13: #10 OR #11 OR #12	1,257,553	
	#3 AND #4 AND #9 AND #13	226	
**Embase**	#1: aged:ab,ti OR 'aged'/exp OR 'older adults'/exp OR 'older adults':ab,ti OR elderly:ab,ti	4,663,857	190
	#2: 'emotion-oriented':ab,ti OR 'emotion-oriented care':ab,ti OR 'reminiscence therapy'/exp OR 'reminiscence therapy':ab,ti OR 'life review therapy'/exp OR 'life review therapy':ab,ti OR 'life review':ab,ti OR 'simulated presence therapy'/exp OR 'simulated presence therapy':ab,ti OR 'validation therapy'/exp OR 'validation therapy':ab,ti OR 'supportive psychotherapy'/exp OR 'supportive psychotherapy':ab,ti–	2,618	
	#3: ('self esteem'/exp OR 'self-esteem':ab,ti OR 'depression'/exp OR depression:ab,ti OR 'life satisfaction'/exp OR 'life satisfaction':ab,ti OR 'loneliness'/exp OR loneliness:ab,ti OR 'cognition'/exp OR cognition:ab,ti) OR 'cognitive function':ab,ti	3,882,670	
	#4: ('randomized controlled trial'/exp OR 'randomized controlled trial':ab,ti OR 'randomised controlled trial':ab,ti OR randomized:ab,ti OR randomised:ab,ti OR 'randomization'/exp OR randomization:ab,ti OR randomisation:ab,ti) AND randomly:ab,ti	1,787,669	
	#1 AND #2 AND #3 AND #4	190	
**OVID-Medline**	1: aged.mp. or Aged/	6,010,135	150
	2: elderly.mp.	311,870	

Databases	Syntaxes	Number	Total
	3: older adults.mp.	123,180	
	4: 1 OR 2 OR 3	6,091,659	
	5: emotion-oriented.mp.	376	
	6:"emotion-oriented care".mp.	18	
	7: "Reminiscence therapy".mp.	340	
	8: "life review".mp.	480	
	9: "Simulated Presence Therapy".mp.	15	
	10: "Validation therapy".mp.	58	
	11: "Supportive psychotherapy".mp.	613	
	12: 5 OR 6 OR 7 OR 8 OR 9 OR10 OR 11	1,829	
	13: self-esteem.mp.	25,979	
	14: Depression/ or depression.mp.	507,504	
	15: "life satisfaction".mp.	11,623	
	16: loneliness.mp. or Loneliness/	14,179	
	17: "cognitive function".mp. or Cognition/	165,431	
	18: 13 OR 14 OR 15 OR 16 OR 17	694,576	
	19: "randomized controlled trial".mp. or Randomized Controlled Trial/	653,142	
	20: "randomised controlled trial".mp.	32,813	
	21: randomized.mp.	1,050,802	
	22: randomised.mp.	135,660	
	23: randomization.mp. or Random Allocation/	148,170	
	24: randomisation.mp.	12,493	

Databases	Syntaxes	Number	Total
	25: randomly.mp.	425,411	
	26: 19 OR 20 OR 21 OR 22 OR 23 OR 24 OR 25	1,420,251	
	4 AND 12 AND 18 AND 26	202	
**PsycINFO**	S1: MA aged OR TI aged OR AB aged OR MA "older adults" OR TI "older adults" OR AB "older adults" OR TI elderly OR AB elderly	702,496	133
	S2: TI "emotion-oriented care" OR AB "emotion-oriented care" OR TI "emotion-oriented" OR AB "emotion-oriented" OR MA "reminiscence therapy" OR TI "reminiscence therapy" OR AB "reminiscence therapy" OR MA "life review therapy" OR TI "life review therapy" OR AB "life review therapy" OR TI "life review" OR AB "life review"	1,535	
	S3: MA "simulated presence therapy" OR TI "simulated presence therapy" OR AB "simulated presence therapy" OR MA "validation therapy" OR TI "validation therapy" OR AB "validation therapy" OR MA "supportive psychotherapy" OR TI "Supportive Psychotherapy" OR AB "Supportive Psychotherapy"	897	
	S4: S2 OR S3	2,418	
	S5: TI "self esteem" OR AB "self esteem" OR MA depression OR TI depression OR AB depression OR TI "life satisfaction" OR AB "life satisfaction" OR MA loneliness OR TI loneliness OR AB loneliness	368,048	
	S6: MA cognition OR TI cognition OR AB cognition OR TI "cognitive function" OR AB "cognitive function"	184,876	
	S7: S5 OR S6	534,766	

Databases	Syntaxes	Number	Total
	S8: MA "randomized controlled trial" OR TI "randomized controlled trial" OR AB "randomized controlled trial" OR TI "randomised controlled trial" OR AB "randomised controlled trial" OR TI randomized OR AB randomized OR TI randomised OR AB randomised OR TI randomization OR AB Randomization	111,175	
	S9: TI Randomisation OR AB Randomisation OR TI randomly OR AB randomly	91,332	
	S10: S8 OR S9	182,510	
	S1 AND S4 and S7 AND S10	133	
**PubMed**	#1: (((aged[MeSH Terms]) OR (aged[Title/Abstract])) OR ("older adults"[Title/Abstract])) OR (Elderly[Title/Abstract])	4,073,959	163
	#2: (((((("emotion-oriented care"[Title/Abstract]) OR ("emotion-oriented"[Title/Abstract])) OR ("Reminiscence Therapy"[Title/Abstract])) OR ("life review"[Title/Abstract])) OR ("Simulated Presence Therapy"[Title/Abstract])) OR ("Validation Therapy"[Title/Abstract])) OR ("Supportive Psychotherapy"[Title/Abstract])	1,838	
	#3: ((((((((("self-esteem"[Title/Abstract]) OR (depression[MeSH Terms])) OR (depression[Title/Abstract])) OR ("life satisfaction"[Title/Abstract]))) OR (loneliness[MeSH Terms])) OR (loneliness[Title/Abstract])) OR (cognition[MeSH Terms])) OR (cognition[Title/Abstract])) OR ("cognitive function"[Title/Abstract])	830,816	
	#4: ((((((("randomized controlled trial"[Title/Abstract])) OR ("randomised controlled trial"[Title/Abstract])) OR (randomized[Title/Abstract])) OR (randomised[Title/Abstract])) OR (randomization[Title/Abstract])) OR (randomisation[Title/Abstract])) OR (randomly[Title/Abstract])	1,162,496	

Databases	Syntaxes	Number	Total
	#5: ((((((aged[MeSH Terms]) OR (aged[Title/Abstract])) OR ("older adults"[Title/Abstract])) OR (Elderly[Title/Abstract])) AND ((((((("emotion-oriented care"[Title/Abstract]) OR ("emotion-oriented"[Title/Abstract])) OR ("Reminiscence Therapy"[Title/Abstract])) OR ("life review"[Title/Abstract])) OR ("Simulated Presence Therapy"[Title/Abstract])) OR ("Validation Therapy"[Title/Abstract])) OR ("Supportive Psychotherapy"[Title/Abstract]))) AND (((((((((("self-esteem"[Title/Abstract]) OR (depression[MeSH Terms])) OR (depression[Title/Abstract])) OR ("life satisfaction"[Title/Abstract]))) OR (loneliness[MeSH Terms])) OR (loneliness[Title/Abstract])) OR (cognition[MeSH Terms])) OR (cognition[Title/Abstract])) OR ("cognitive function"[Title/Abstract]))) AND (((((((("randomized controlled trial"[Title/Abstract])) OR ("randomised controlled trial"[Title/Abstract])) OR (randomized[Title/Abstract])) OR (randomised[Title/Abstract])) OR (randomization[Title/Abstract])) OR (randomisation[Title/Abstract])) OR (randomly[Title/Abstract]))	163	
**Scopus**	1: (TITLE-ABS-KEY (aged) OR TITLE-ABS-KEY ("older adults") OR TITLE-ABS-KEY (elderly))	6,505,385	277
	2: (TITLE-ABS-KEY ("emotion-oriented care") OR TITLE-ABS-KEY ("emotion-oriented") OR TITLE-ABS-KEY ("reminiscence therapy") OR TITLE-ABS-KEY ("life review") OR TITLE-ABS-KEY ("simulated presence therapy") OR TITLE-ABS-KEY ("validation therapy") OR TITLE-ABS-KEY ("supportive psychotherapy"))	3,464	

Databases	Syntaxes	Number	Total
	3: (TITLE-ABS-KEY ("self-esteem") OR TITLE-ABS-KEY (depression) OR TITLE-ABS-KEY ("life satisfaction") OR TITLE-ABS-KEY (loneliness) OR TITLE-ABS-KEY ("cognitive function") OR TITLE-ABS-KEY (cognition))	1,455,890	
	4: (TITLE-ABS-KEY ("randomized controlled trial") OR TITLE-ABS-KEY ("randomised controlled trial") OR TITLE-ABS-KEY (randomized) OR TITLE-ABS-KEY (randomised) OR TITLE-ABS-KEY (randomization) OR TITLE-ABS-KEY (randomisation) OR TITLE-ABS-KEY (randomly))	1,965,408	
	5: ((TITLE-ABS-KEY (aged) OR TITLE-ABS-KEY ("older adults") OR TITLE-ABS-KEY (elderly))) AND ((TITLE-ABS-KEY ("emotion-oriented care") OR TITLE-ABS-KEY ("emotion-oriented") OR TITLE-ABS-KEY ("reminiscence therapy") OR TITLE-ABS-KEY ("life review") OR TITLE-ABS-KEY ("simulated presence therapy") OR TITLE-ABS-KEY ("validation therapy") OR TITLE-ABS-KEY ("Supportive Psychotherapy"))) AND ((TITLE-ABS-KEY (self-esteem) OR TITLE-ABS-KEY (depression) OR TITLE-ABS-KEY ("life satisfaction") OR TITLE-ABS-KEY (loneliness) OR TITLE-ABS-KEY ("cognitive function") OR TITLE-ABS-KEY (cognition))) AND ((TITLE-ABS-KEY ("randomized controlled trial") OR TITLE-ABS-KEY ("randomised controlled trial") OR TITLE-ABS-KEY (randomized) OR TITLE-ABS-KEY (randomised) OR TITLE-ABS-KEY (randomization) OR TITLE-ABS-KEY (randomisation) OR TITLE-ABS-KEY (randomly)))	277	
**Web of Science**	1: aged (Title) or aged (Abstract) or elderly (Title) or elderly (Abstract) or "older adults" (Title) or "older adults" (Abstract)	3,690,103	112

Databases	Syntaxes	Number	Total
	2: emotion-oriented (Title) or emotion-oriented (Abstract) or "emotion-oriented care" (Title) or "emotion-oriented care" (Abstract) or "reminiscence therapy" (Title) or "reminiscence therapy" (Abstract) or "life review" (Title) or "life review" (Abstract) or "simulated presence therapy" (Title) or "simulated presence therapy" (Abstract) or "validation therapy" (Title) or "validation therapy" (Abstract) or "Supportive Psychotherapy" (Title) or "Supportive Psychotherapy" (Abstract)	1,816	
	3: self-esteem (Title) or self-esteem (Abstract) or depression (Title) or depression (Abstract) or "life satisfaction" (Title) or "life satisfaction" (Abstract) or loneliness (Title) or loneliness (Abstract) or "cognitive function" (Title) or "cognitive function" (Abstract) or cognition (Title) or cognition (Abstract)	653,545	
	4: "randomized controlled trial" (Title) or "randomized controlled trial" (Abstract) or "randomised controlled trial" (Title) or "randomised controlled trial" (Abstract) or randomized (Title) or randomized (Abstract) or randomised (Title) or randomised (Abstract) or randomization (Title) or randomization (Abstract) or randomisation (Title) or randomisation (Abstract) or randomly (Title) or randomly (Abstract)	1,234,396	
	5: #1 AND #2 AND #3 AND #4	112	
		**TOTAL**	**1430**

Table S3. List of the included studies.

Databases

1. Afonso R, Bueno B. Effects of a reminiscence program on depressive symptomatology in an elderly population in Portugal. Revista Espanola de Geriatria y Gerontologia. 2009;44:317-22.
2. Aydogdu O, Tastan S, Kublay G. The effects of the instrumental reminiscence therapy based on Roy's adaptation model on adaptation, life satisfaction and happiness in older people: A randomized controlled trial. International Journal of Nursing Practice (John Wiley & Sons, Inc). 2023;29:1-9.
3. Bazrafshan M-R, Faramarzian Z, Jokar M, Delam H, Mansouri A, Soufi O, et al. The Effect of Reminiscence on Depression in Elderly People with Suicidal Ideation: A Randomized Controlled Trial. Jundishapur Journal of Chronic Disease Care (JJCDC). 2022;11:1-9.
4. Bazrafshan M-R, Jokar M, Soufi O, Delam H. The effect of structured group reminiscence on depression and anxiety of the elderly female hookah users. Journal of Substance Use. 2022;27:528-34.
5. Chan MF, Ng SE, Tien A, Man Ho RC, Thayala J. A randomised controlled study to explore the effect of life story review on depression in older Chinese in Singapore. Health & Social Care in the Community. 2013;21:545-53.
6. Chiang K, Chu H, Chang H, Chung M, Chen C, Chiou H, et al. The effects of reminiscence therapy on psychological well-being, depression, and loneliness among the institutionalized aged. International Journal of Geriatric Psychiatry. 2010;25:380-8.
7. Chippendale T. The effects of life review through writing on depressive symptoms and life satisfaction in older adults: ProQuest Information & Learning; 2012.
8. Cook EA. Effects of reminiscence on life satisfaction of elderly female nursing home residents. Health Care for Women International. 1998;19:109-18.
9. Duru Aşiret G, Dutkun M. The effect of reminiscence therapy on the adaptation of elderly women to old age: A randomized clinical trial. Complementary Therapies in Medicine. 2018;41:124-9.
10. Gonçalves DC, Albuquerque PB, Paul C. Life review with older women: an intervention to reduce depression and improve autobiographical memory. Aging Clinical & Experimental Research. 2009;21:369-71.
11. Hsieh CJ, Chang C, Su SF, Hsiao YL, Shih YW, Han WH, et al. Reminiscence group therapy on depression and apathy in nursing home residents with mild-to-moderate dementia. 2010;2:72-8.
12. Ilali ES, Mokhtary F, Mousavinasab N, Tirgari AH. Impact of Art-Based Life Review on Depression Symptoms Among Older Adults. Art Therapy: Journal of the American Art Therapy Association. 2018;35:148-55.
13. Justo-Henriques SI, Pérez-Sáez E, Alves Apóstolo JL. Multicentre randomised controlled trial about the effect of individual reminiscence therapy in older adults with neurocognitive disorders. Int J Geriatr Psychiatry. 2021;36:704-12.

14. Justo-Henriques SI, Pérez-Sáez E, Apóstolo JLA, Carvalho JO. Effectiveness of a Randomized Controlled Trial of Individual Reminiscence Therapy on Cognition, Mood and Quality of Life in Azorean Older Adults with Neurocognitive Disorders. J Clin Med. 2021;10.
15. Keisari S, Palgi Y, Yaniv D, Gesser-Edelsburg A. Participation in Life-Review Playback Theater Enhances Mental Health of Community-Dwelling Older Adults: A Randomized Controlled Trial. 2022;16:302-17.
16. Lan X, Xiao H, Chen Y. Life review for Chinese older adults in nursing homes: cultural acceptance and its effects. International Psychogeriatrics. 2019;31:527-35.
17. Lan X, Xiao H, Chen Y, Zhang X. Effects of Life Review Intervention on Life Satisfaction and Personal Meaning Among Older Adults With Frailty. Journal of Psychosocial Nursing & Mental Health Services. 2018;56:30-6.
18. Li M, Lyu J-h, Zhang Y, Gao M-l, Li R, Mao P-x, et al. Efficacy of Group Reminiscence Therapy on Cognition, Depression, Neuropsychiatric Symptoms, and Activities of Daily Living for Patients With Alzheimer Disease. Journal of Geriatric Psychiatry & Neurology. 2020;33:272-81.
19. Li S, Dai Y, Zhou Y, Zhang J, Zhou C. Efficacy of group reminiscence therapy based on Chinese traditional festival activities (ctfa-grt) on loneliness and perceived stress of rural older adults living alone in China: A randomized controlled trial. Aging & Mental Health. 2022.
20. Mastel-Smith BA, McFarlane J, Sierpina M, Malecha A, Haile B. Geropsychiatry. Improving depressive symptoms in community-dwelling older adults. Journal of Gerontological Nursing. 2007;33:13-9.
21. Musavi M, Mohammadian S, Mohammadinezhad B. The effect of group integrative reminiscence therapy on mental health among older women living in Iranian nursing homes. Nursing Open. 2017;4:303-9.
22. Nomura N. [Individual reminiscence therapy improves self-esteem for Japanese community-dwelling older adults]. Shinrigaku Kenkyu. 2009;80:42-7.
23. Pérez-Sáez E, Justo-Henriques SI, Alves Apóstolo JL. Multicenter randomized controlled trial of the effects of individual reminiscence therapy on cognition, depression and quality of life: Analysis of a sample of older adults with Alzheimer's disease and vascular dementia. Clin Neuropsychol. 2022;36:1975-96.
24. Pishvaei M, Moghanloo RA, Moghanloo VA. The Efficacy of Treatment Reminders of Life with Emphasis on Integrative Reminiscence on Self-Esteem and Anxiety in Widowed Old Men. Iranian Journal of Psychiatry. 2015;10:19-24.
25. Preschl B, Maercker A, Wagner B, Forstmeier S, Baños RM, Alcañiz M, et al. Life-review therapy with computer supplements for depression in the elderly: A randomized controlled trial. Aging & Mental Health. 2012;16:964-74.
26. Rob SS, Stegman CE, Wolanin MO. No research versus research with compromised results: A study of validation therapy. Nursing Research. 1986;35:113-8.
27. Serrano JP, Latorre JM, Gatz M, Montanes J. Life review therapy using autobiographical retrieval practice for older adults with depressive symptomatology. Psychol Aging. 2004;19:270-7.

28. Stevens-Ratchford RG. The effect of life review reminiscence activities on depression and self-esteem in older adults. American Journal of Occupational Therapy. 1993;47:413-20.
29. Subramaniam P, Woods B, Whitaker C. Life review and life story books for people with mild to moderate dementia: a randomised controlled trial. Aging & mental health. 2014;18:363-75.
30. Van Bogaert P, Tolson D, Eerlingen R, Carvers D, Wouters K, Paque K, et al. SolCos model-based individual reminiscence for older adults with mild to moderate dementia in nursing homes: A randomized controlled intervention study. Journal of Psychiatric and Mental Health Nursing. 2016;23:568-75.
31. Wang JJ. Group reminiscence therapy function of demented elderly for cognitive and affective in Taiwan. International Journal of Geriatric Psychiatry. 2007;22:1235-40.
32. Wu LF, Koo M. Randomized controlled trial of a six-week spiritual reminiscence intervention on hope, life satisfaction, and spiritual well-being in elderly with mild and moderate dementia. International Journal of Geriatric Psychiatry. 2016;31:120-7.

Manual Search

33. Balci AS, Kolaç N, Melisa K, İbrahim S, Düzen KÖ. Effect of Group Reminiscence Therapy on the Loneliness, Depression and Life Satisfaction of the Elderly in the Nursing Homes. Acıbadem Üniversitesi Sağlık Bilimleri Dergisi. 2022;13:394-9.

Citation Searching

34. Choy JC, Lou VW. Effectiveness of the modified instrumental reminiscence intervention on psychological well-being among community-dwelling Chinese older adults: A randomized controlled trial. The American Journal of Geriatric Psychiatry. 2016;24:60-9.
35. Deponte A, Missan R. Effectiveness of validation therapy (VT) in group: preliminary results. Archives of gerontology and geriatrics. 2007;44:113-7.
36. Hojjati H, Sharifnia S, Hassanalipour S, Akhonzadeh G, Asayesh H. The effect of reminiscence on the amount of group self-esteem and life satisfaction of the elderly. Journal of Urmia Nursing & Midwifery Faculty. 2011;9.
37. Jahanbin I, Mohammadnejad S, Sharif F. The effect of group reminiscence on the cognitive status of elderly people supported by ilam welfare organization in 2013; a randomized controlled clinical trial. Int J Community Based Nurs Midwifery. 2014;2:231-9.

Table S4. Characteristics of the Included Studies.

No.	Author (year)<br>Location	Participants' characteristics<br>Demographic	 Diagnosis	Interventions<br>Procedure	 Outcomes/ assessment tools	 Follow-up
1.	Afonso<br>(2009)<br>Portugal	Sample size<br>Overall: 60<br>EG: 30<br>CG: 30<br>Age:<br>65-94<br>Gender:<br>M: 10 (16.7%)<br>F: 50 (83.3%)<br>Setting:<br>Community	Type:<br>Depression<br>Duration:<br>NA<br>Diagnosis tool:<br>NA<br>Severity:<br>NA	Experiment:<br>Semi-structured reminiscence program<br>Frequency: 1x /per week<br>Duration: NI<br>Total sessions: 5<br>Total week: 9<br>Format: individual<br>Control:<br>CG: No intervention	Psychological:<br>Depression (CES-D)	- Baseline<br>- Post-test (9 weeks)
2.	Aydogdu<br>(2022)<br>Turkey	Sample size<br>Overall: 34<br>EG: 17<br>CG: 17<br>Mean (SD) age:<br>EG: 76.2±9.2<br>CG: 73.9±7.4<br>Gender:<br>M: 16 (47%)<br>F: 18 (53%)<br>Setting:<br>Institution	Type:<br>No cognitive impairment<br>Duration:<br>NA<br>Diagnosis tool:<br>MMSE<br>Severity:<br>NA	Experiment:<br>Reminiscence therapy<br>Frequency: 1x /per week<br>Duration: 45-60 mins<br>Total sessions: 8<br>Total week: 8<br>Format: group<br>Control:<br>Usual care	Psychological:<br>Life satisfaction (LSI-A)	- Baseline<br>- Post-test (8 weeks)
3.	Balci et al.<br>(2022)<br>Turkey	Sample size<br>Overall: 76<br>EG: 37<br>CG: 39<br>Mean (SD) age:<br>NI<br>Gender:<br>M: 49 (64.5%)<br>F: 27 (35.5%)<br>Setting:<br>Institution	Type:<br>no mental illness<br>Duration:<br>NA<br>Diagnosis tool:<br>NA<br>Severity:<br>NA	Experiment:<br>Reminiscence intervention<br>Frequency: 1x/per week<br>Duration: 30-45 mins<br>Total sessions: 6<br>Total week: 6<br>Format: group<br>Control:<br>No intervention	Psychological:<br>Depression (BDS)<br>Life satisfaction (LSS)<br>Loneliness (UCLA)	- Baseline<br>- Post-test (6 weeks)
4.	Bazrafshan<br>(2022a)<br>Iran	Sample size<br>Overall: 30<br>EG: 15	Type:<br>Depression<br>Duration:	Experiment:<br>Reminiscence<br>Frequency: 2x /per week	Psychological:<br>Depression (GDS)	- Baseline<br>- Post-test (4 weeks)<br>- Follow-up (4weeks)

		CG: 15<br>Mean (SD) age:<br>EG: 67.9±6.1<br>CG: 69.0±5.5<br>Gender:<br>M: 16 (53.3%)<br>F: 14 (46.7%)<br>Setting:<br>Institution	NA<br>Diagnosis tool:<br>GDS<br>Severity:<br>Mild to Moderate	Duration: 30 mins<br>Total sessions: 8<br>Total week: 4<br>Total time: 4 hours<br>Format: group<br>Control:<br>Usual care		
5.	Bazrafshan<br>(2022b)<br>Iran	Sample size<br>Overall: 24<br>EG: 12<br>CG: 12<br>Mean (SD) age:<br>EG: 69.8±5.0<br>CG: 70.1±5.4<br>Gender:<br>M: 12 (50%)<br>F: 12 (50%)<br>Setting:<br>Community	Type:<br>1. Depression<br>2. Anxiety<br>Duration:<br>NA<br>Diagnosis tool:<br>1. ED-5Q<br>2. Spielberger Anxiety Inventory (state anxiety, trait anxiety)<br>Severity:<br>1. Mild to Moderate<br>2. Moderate to moderately severe (state anxiety), moderate (trait anxiety)	Experiment:<br>Reminiscence<br>Frequency: 1x /per week<br>Duration: 60-90 mins<br>Total sessions: 12<br>Total week: 12<br>Format: group<br>Control:<br>Usual care	Psychological:<br>Depression (EDI)	- Baseline<br>- Post-test (12 weeks)<br>- Follow-up (4 weeks)
6.	Chan et al.<br>(2013)<br>Singapore	Sample size<br>Overall: 26<br>EG: 14<br>CG: 12<br>Mean (SD) age:<br>EG: 70.4±7.1<br>CG: 68.9±6.7<br>Gender:<br>M: 5 (19.2%)<br>F: 21(80.8%)<br>Setting:<br>Community	Type:<br>Depression<br>Duration:<br>NA<br>Diagnosis tool:<br>GDS-15<br>Severity:<br>Mild to severe	Experiment:<br>Life story review<br>Frequency: 1x/per week<br>Duration: 30-45 mins<br>Total sessions: 4<br>Total week: 4<br>Total time: 3 hours<br>Format: individual<br>Control:<br>Usual care	Psychological:<br>Depression (GDS-15)	- Baseline<br>- During process (2, 3 weeks)<br>- Post-test (4 weeks)<br>- Follow-up (8 weeks)
7.	Chiang et al.<br>(2010)<br>Taiwan	Sample size<br>Overall: 92<br>EG: 45<br>CG: 47	Type:<br>Cognitive impairment<br>Duration:<br>NA	Experiment:<br>Reminiscence therapy<br>Frequency: 1x/per week<br>Duration: 90 mins	Psychological:<br>Depression (CES-D)<br>Loneliness (RULS-V3)	- Baseline<br>- Post-test (8 weeks)<br>- Follow-up (12 weeks)

		Mean (SD) age:<br>EG: 77.4±3.7<br>CG: 77.1±4.2<br>Gender:<br>NI<br>Setting:<br>Institution	Diagnosis tool:<br>MMSE<br>Severity:<br>Normal to mild	Total sessions: 8<br>Total week: 8<br>Format: group<br>Control:<br>Usual care		
8.	Chippendale (2012) USA	Sample size<br>Overall: 45<br>EG: 23<br>CG: 22<br>Mean (SD) age:<br>84.0±7.56<br>Gender:<br>M: 14 (31.1%)<br>F: 31(68.9%)<br>Setting:<br>Institution	Type:<br>Depression<br>Duration:<br>NA<br>Diagnosis tool:<br>GDS<br>Severity:<br>Mild to severe	Experiment:<br>Life review writing<br>Frequency: 1x/per week<br>Duration: 90 mins<br>Total sessions: 8<br>Total week: 8<br>Format: group<br>Control:<br>Wait-list	Psychological:<br>Depression (GDS-15)<br>Life satisfaction (LSI-A)	- Baseline<br>- Post-test (8 weeks)
9.	Choy (2016) China	Sample size<br>Overall: 114<br>EG: 46<br>CG: 68<br>Mean (SD) age:<br>EG: 77.2±7.6<br>CG: 79.7±6.7<br>Gender:<br>M: 40 (35.1%)<br>F: 74 (64.9%)<br>Setting:<br>Community	Type:<br>Depression<br>Duration:<br>NA<br>Diagnosis tool:<br>GDS-15<br>Severity:<br>Mild to moderate	Experiment:<br>Instrumental reminiscence intervention<br>Frequency: 1x/per week<br>Duration: 90 mins<br>Total sessions: 6<br>Total week: 6<br>Format: group<br>Control:<br>Wait-list	Psychological:<br>Depression (GDS-15)<br>General outcomes:<br>Life satisfaction (LSS-C)	- Baseline<br>- Post-test (6 weeks)<br>- 1st follow-up (2 weeks)<br>- 2nd follow-up (6 weeks)
10.	Cook (1998) USA	Sample size<br>Overall: 36<br>EG: 18<br>CG: 18<br>Mean (SD) age:<br>82.4 (NI)<br>Gender:<br>M: 0 (0%)<br>F: 36 (100%)<br>Setting:<br>Institution	Type:<br>Physical health<br>Duration:<br>NA<br>Diagnosis tool:<br>NA<br>Severity:<br>NA	Experiment:<br>Reminiscence<br>Frequency: 1x/per week<br>Duration: 60 mins<br>Total sessions: 16<br>Total week: 16<br>Format: group<br>Control:<br>No treatment	Psychological:<br>Life satisfaction (LISA)	- Baseline<br>- Post-test (16 weeks)

11.	Deponte<br>(2007)<br>Italy	Sample size<br>Overall: 27<br>EG1: 9<br>EG2: 9<br>CG: 9<br>Mean (SD) age:<br>86.8(NI)<br>Gender:<br>NI<br>Setting:<br>Institution	Type:<br>Dementia<br>Type of dementia:<br>NA<br>Duration:<br>NA<br>Diagnosis tool:<br>NA<br>Severity:<br>NA	Experiment:<br>EG1: Validation therapy<br>EG2: Sensorial reminiscence<br>Frequency: 2x /per week<br>Duration: 45-60 mins<br>Total sessions: NI<br>Total week: 12<br>Format: group<br>Control:<br>Usual care	Global cognitive function:<br>Cognitive function (MMSE)	- Baseline<br>- Post-test (12 weeks)
12.	Duru Aşiret<br>(2018)<br>Turkey	Sample size<br>Overall: 56<br>EG: 30<br>CG: 26<br>Mean (SD) age:<br>72.2±6.3<br>Gender:<br>M: 0 (0%)<br>F: 50 (100%)<br>Setting:<br>Community	Type:<br>Psychological health<br>Duration:<br>NA<br>Diagnosis tool:<br>MMSE<br>Severity:<br>Normal	Experiment:<br>Reminiscence therapy<br>Frequency: 1x/per week<br>Duration: 35-45 mins<br>Total sessions: 8<br>Total week: 8<br>Format: individual<br>Control:<br>No intervention	Global cognitive function:<br>Cognitive function (MMSE)	- Baseline<br>- Post-test (8 weeks)
13.	Goncalves et al.<br>(2009)<br>Portugal	Sample size<br>Overall: 22<br>EG: 11<br>CG: 11<br>Mean (SD) age:<br>80.7±4.5<br>Gender:<br>M: 0 (0%)<br>F: 22 (100%)<br>Setting:<br>Institution	Type:<br>Depression<br>Duration:<br>NA<br>Diagnosis tool:<br>GDS<br>Severity:<br>Mild to severe	Experiment:<br>Life review<br>Frequency: 2x/per week<br>Duration: 60 mins<br>Total sessions: 4<br>Total week: 2<br>Format: group<br>Control:<br>Wait-list	Psychological:<br>Depression (GDS)<br>Life satisfaction (LISA)	- Baseline<br>- Post-test (4 weeks)
14.	Hojjati<br>(2011)<br>Iran	Sample size<br>Overall: 32<br>EG: 16<br>CG:16<br>Mean (SD) age:<br>NI<br>Gender:<br>NI<br>Setting:	Type:<br>Normal cognitive function<br>Duration:<br>NA<br>Diagnosis tool:<br>NA<br>Severity:<br>NA	Experiment:<br>Reminiscence<br>Frequency: 2x/per week<br>Duration: 60-90 mins<br>Total sessions: 8<br>Total week: 4<br>Format: group<br>Control:<br>No treatment	Psychological:<br>Self-esteem (RSES)<br>Life satisfaction (LISA)	- Baseline<br>- Post-test (4 weeks)

		Institution				
15.	Hsieh<br>(2010)<br>Taiwan	Sample size<br>Overall: 61<br>EG: 29<br>CG: 32<br>Mean (SD) age:<br>EG: 77.9±5.6<br>CG: 77.3±10.5<br>Gender:<br>M: 36 (59%)<br>F: 25 (41%)<br>Setting:<br>Institution	Type:<br>Dementia<br>Type of dementia:<br>AD<br>Duration:<br>NA<br>Diagnosis tool:<br>MMSE<br>CDS<br>Severity:<br>mild-to-moderate	Experiment:<br>Reminiscence therapy<br>Frequency: 1x/per week<br>Duration: 40-50 mins<br>Total sessions: 12<br>Total week: 12<br>Format: group<br>Control:<br>No treatment	Psychological:<br>Depression (GDS)	- Baseline<br>- Post-test (12 weeks)
16.	Ilali<br>(2019)<br>Iran	Sample size<br>Overall: 54<br>EG: 27<br>CG:27<br>Mean (SD) age:<br>70 (NI)<br>Gender:<br>M: 19 (35.2%)<br>F: 35 (64.8%)<br>Setting:<br>Institution	Type:<br>Depression<br>Diagnosis tool:<br>GDS<br>Severity:<br>Normal to Moderate	Experiment:<br>Art-based Life review<br>Frequency: 1x/per week<br>Duration: 60 mins<br>Total sessions: 6<br>Total week: 6<br>Total time: 6 hours<br>Format: group<br>Control:<br>No intervention, daily routine	Psychological:<br>Depression (GDS-15)	- Baseline<br>- Post-test (7 weeks)<br>- Follow-up (4 weeks)
17.	Jahanbin<br>(2014)<br>Iran	Sample size<br>Overall: 100<br>EG: 50<br>CG: 50<br>Mean (SD) age:<br>71±8.1<br>Gender:<br>M: 54 (54%)<br>F: 46 (46%)<br>Setting:<br>Community	Type:<br>Cognitive impairments<br>Duration:<br>NA<br>Diagnosis tool:<br>MMSE<br>Severity:<br>Mild to moderate	Experiment:<br>Reminiscence program<br>Frequency: 2x/per week<br>Duration: 60 mins<br>Total sessions: 16<br>Total week: 8<br>Format: group<br>Control:<br>No intervention	Global cognitive function:<br>Cognitive function (MMSE)	- Baseline<br>- Post-test (8 weeks)<br>- 1st Follow-up (8 weeks)<br>- 2nd Follow-up (12 weeks)
18.	Justo-Henriques et al.<br>(2021a)<br>Portugal	Sample size<br>Overall: 251<br>EG: 131<br>CG: 120<br>Mean (SD) age:<br>EG: 82.6±7.2<br>CG: 82.9±7.2	Type:<br>Neurocognitive disorders<br>Type of Neurocognitive disorders:<br>AD<br>PD	Experiment:<br>Reminiscence treatment<br>Frequency: 2x/per week<br>Duration: 50 mins<br>Total sessions: 13<br>Total week: 13<br>Format: individual	Psychological:<br>Depression (GDS-15)<br>Global cognitive function:<br>Cognitive function (MMSE)	- Baseline<br>- Post-test (15 weeks)

		Gender:<br>M: 69 (27.5%)<br>F: 182 (72.5%)<br>Setting:<br>Community	Frontotemporal degeneration<br>VD<br>Traumatic brain injury<br>Others<br>Duration:<br>NA<br>Diagnosis tool:<br>DSM-V<br>Severity:<br>NA	Control:<br>Usual treatment		
19.	Justo-Henriques et al. (2021b)<br>Portugal	Sample size<br>Overall: 122<br>EG: 62<br>CG: 60<br>Mean (SD) age:<br>EG: 80.8±6.7<br>CG: 79.6±7.4<br>Gender:<br>M: 32(26.2%)<br>F: 90 (73.8%)<br>Setting:<br>Community	Type:<br>Neurocognitive disorders<br>Type of Neurocognitive disorders:<br>AD<br>PD<br>Frontotemporal degeneration<br>VD<br>Traumatic brain injury<br>Others<br>Duration:<br>NA<br>Diagnosis tool:<br>DSM-V<br>Severity:<br>NA	Experiment:<br>Reminiscence treatment<br>Frequency: 2x/per week<br>Duration: 50 mins<br>Total sessions: 13<br>Total week: 13<br>Format: individual<br>Control:<br>Usual treatment	Psychological:<br>Depression (GDS-15)<br>Global cognitive function:<br>Cognitive function (MMSE)	- Baseline<br>- Post-test (15 weeks)
20.	Keisari (2022)<br>Israel	Sample size<br>Overall: 78<br>EG: 40<br>CG:38<br>Mean (SD) age:<br>EG: 78.7±6.9<br>CG: 80.6±6.8<br>Gender:<br>M: 15 (19.2%)<br>F: 63 (80.8%)<br>Setting:<br>Community	Type:<br>Normal cognitive level<br>Diagnosis tool:<br>MMSE<br>Severity:<br>NA	Experiment:<br>Life review theater<br>Frequency: 1x/per week<br>Duration: 90 mins<br>Total sessions: 12<br>Total week: 12<br>Format: group<br>Control:<br>Care as usual	Psychological:<br>Self-esteem (RSES)<br>Depression (GDS)<br>Loneliness (UCLA)<br>Life satisfaction (SLS)	- Baseline<br>- Post-test (12 weeks)<br>- Follow-up (12 weeks)
21.	Lan et al.	Sample size	Type:	Experiment:	Psychological:	- Baseline

	(2019)<br>China	Overall: 62<br>EG: 31<br>CG: 31<br>Mean (SD) age:<br>EG: 83.1±6.9<br>CG: 82.3±7.1<br>Gender:<br>M: 23 (37%)<br>F: 39 (63%)<br>Setting:<br>Institution	Normal cognitive level<br>Duration:<br>NA<br>Diagnosis tool:<br>NA<br>Severity:<br>NA	Life review<br>Frequency: 1x/per week<br>Duration: 60 mins<br>Total sessions: 6<br>Total week: 6<br>Total time: 6 hours<br>Format: group<br>Control:<br>Usual care	Self-esteem (RSES)<br>Depression (GDS)	- Post-test (6 weeks)
22.	Lan et al.<br>(2018)<br>China	Sample size<br>Overall: 74<br>EG: 37<br>CG: 37<br>Mean (SD) age:<br>EG: 83.1±6.5<br>CG: 83.5±6.6<br>Gender:<br>M: 26 (35.1%)<br>F: 48 (64.9%)<br>Setting:<br>Institution	Type:<br>Normal cognitive level<br>Duration:<br>NA<br>Diagnosis tool:<br>NA<br>Severity:<br>NA	Experiment:<br>Life review intervention<br>Frequency: 1x/per week<br>Duration: 30-60 mins<br>Total sessions: 6<br>Total week: 6<br>Total time: 3-6 hours<br>Format: group<br>Control:<br>Usual care	General outcomes:<br>Life satisfaction (LSIA)	- Baseline<br>- Post-test (6 weeks)
23.	Li et al.<br>(2020)<br>China	Sample size<br>Overall: 85<br>EG: 43<br>CG: 42<br>Mean (SD) age:<br>83.4±6.1<br>Gender:<br>M: 47 (55.3%)<br>F: 38 (44.7%)<br>Setting:<br>Institution	Type:<br>Neurodegenerative disease<br>Type of Neurodegenerative disease:<br>AD<br>Duration:<br>NA<br>Diagnosis tool:<br>NINDS<br>ADRDS<br>CDR<br>Severity:<br>Mild to moderate	Experiment:<br>Reminiscence therapy<br>Frequency: 2x/per week<br>Duration: 30-45 mins<br>Total sessions: 24<br>Total week: 12<br>Total time: 12-18 hours<br>Format: group<br>Control:<br>Usual care	Psychological<br>Depression (CSDD)<br>Global cognitive function:<br>Cognitive function (ADAS-CO)	- Baseline<br>- During-test (4 weeks)<br>- Post-test (12 weeks)<br>- Follow-up (12 weeks)
24.	Li et al.<br>(2022)<br>China	Sample size<br>Overall: 60<br>EG: 30<br>CG: 30	Type:<br>No mental illness<br>Duration:<br>NA	Experiment:<br>Reminiscence therapy based on Chinese traditional festival activities	Social function:<br>Loneliness (UCLA-LA)	- Baseline<br>- Post-test (32 weeks)<br>- Follow-up (12 weeks)

		Mean (SD) age:<br>65.7±3.7<br>Gender:<br>M: 22 (36.7%)<br>F: 38 (63.3%)<br>Setting:<br>Community	Diagnosis tool:<br>NA<br>Severity:<br>NA	Frequency: 1x/per month<br>Duration: 240 mins<br>Total sessions: 8<br>Total week: 8<br>Format: group<br>Control:<br>Usual care		
25.	Mastel-Smith et al. (2007)<br>USA	Sample size<br>Overall: 31<br>EG: 15<br>CG: 16<br>Mean (SD) age:<br>EG: 70.1±6.8<br>CG: 72.7±10.4<br>Gender:<br>M: 6 (19%)<br>F: 25 (81%)<br>Setting:<br>Community	Type:<br>Depression<br>Duration:<br>NA<br>Diagnosis tool:<br>NA<br>Severity:<br>NA	Experiment:<br>Life review<br>Frequency: 1x/per week<br>Duration: 120 mins<br>Total sessions: 10<br>Total week: 10<br>Format: group<br>Control:<br>Wait-list	Psychological<br>Depression (BSI 18)	- Baseline<br>- Post-test (10 weeks)
26.	Musavi et al. (2013)<br>Iran	Sample size<br>Overall: 84<br>EG: 42<br>CG: 42<br>Age:<br>>80<br>Gender:<br>M: 6 (7.1%)<br>F: 78 (92.9%)<br>Setting:<br>Institution	Type:<br>Depression<br>Duration:<br>NA<br>Diagnosis tool:<br>GDS<br>Severity:<br>NA	Experiment:<br>Reminiscence therapy.<br>Frequency: 2x/per week<br>Duration: 90mins<br>Total sessions: 8<br>Total week: 4<br>Format: group<br>Control:<br>Usual care	Psychological<br>Depression (GDS)	- Baseline<br>- Post-test (8 weeks)
27.	Nomura (2009)<br>Japan	Sample size<br>Overall: 80<br>EG: 40<br>CG: 40<br>Mean (SD) age:<br>EG: 82.9±5.6<br>CG: 82.2±6.5<br>Gender:<br>M: 23 (28.8%)<br>F: 57 (71.2%)<br>Setting:	Type:<br>No mental illness, no dementia<br>Duration:<br>NA<br>Diagnosis tool:<br>NA<br>Severity:<br>NA	Experiment:<br>Reminiscence therapy<br>Frequency: 1x/per week<br>Duration: 30-60mins<br>Total sessions: 5-6<br>Total week: 5-6<br>Format: individual<br>Control:<br>Usual care	Psychological:<br>Self-esteem (RSES)<br>Depression (GDS)<br>Life satisfaction (LISA)	- Baseline<br>- Post-test (6 weeks)

		Community				
28.	Pérez-Sáez et al.<br>(2022)<br>Portugal	Sample size<br>Overall: 148<br>EG: 74<br>CG: 74<br>Mean (SD) age:<br>EG: 82.4±7.6<br>CG: 82.7±7.3<br>Gender:<br>M: 44 (29.7%)<br>F: 104 (70.3%)<br>Setting:<br>Community and institution	Type:<br>Neurocognitive disorder<br>Type of neurocognitive disorder:<br>AD<br>VD<br>Duration:<br>NA<br>Diagnosis tool:<br>DSM-IV<br>Severity:<br>NA	Experiment:<br>Reminiscence therapy Frequency:<br>2x/per week<br>Duration: 50mins<br>Total sessions: 26<br>Total week: 13<br>Format: individual<br>Control:<br>Usual care	Psychological<br>Depression (GDS-15)<br>Global cognitive function:<br>Cognitive function (MMSE)	- Baseline<br>- Post-test (15 weeks)
29.	Pishvaei et al.<br>(2015)<br>Iran	Sample size<br>Overall: 34<br>EG: 17<br>CG: 17<br>Mean (SD) age:<br>EG: 69.4±5.7<br>CG: 70.0±5.4<br>Gender:<br>M: 34 (100%)<br>F: 0 (0%)<br>Setting:<br>Community	Type:<br>Not having memory impairment<br>Duration:<br>NA<br>Diagnosis tool:<br>NA<br>Severity:<br>NA	Experiment:<br>Integrative reminiscence<br>Frequency: 1x/per week<br>Duration: 60mins<br>Total sessions: 6<br>Total week: 6<br>Format: group<br>Control:<br>Usual care	Psychological:<br>Self-esteem (RSES)	- Baseline<br>- Post-test (6 weeks)
30.	Preschl et al.<br>(2012)<br>German	Sample size<br>Overall: 36<br>EG: 20<br>CG: 16<br>Mean (SD) age:<br>EG: 72.5±4.5<br>CG: 67.0±3.1<br>Gender:<br>M: 12 (33.3%)<br>F: 24 (66.7%)<br>Setting:<br>Community	Type:<br>Depression<br>Duration:<br>NA<br>Diagnosis tool:<br>NA<br>Severity:<br>Mild to moderate	Experiment:<br>Life review therapy<br>Frequency: 1x/per week<br>Duration: 60-90mins<br>Total sessions: 6<br>Total week: 6<br>Format: group<br>Control:<br>Wait-list	Psychological:<br>Self-esteem (RSES)<br>Depression (BDI-II)<br>Life satisfaction (LSIA)	- Baseline<br>- Post-test (6 weeks)<br>- Follow up (12 weeks) only for EG
31.	Robb et al.<br>(1986)<br>USA	Sample size<br>Overall: 36<br>EG: 20<br>CG: 16	Type:<br>disorientated, with a dementia<br>Duration:	Experiment:<br>Validation therapy<br>Frequency: 2x/per week<br>Duration: NI	Global cognitive function:<br>Cognitive function (MSQ)	- Baseline<br>- Post-test (36 weeks)

		Mean (SD) age:<br>EG: 81(NI)<br>CG: 80(NI)<br>Gender:<br>M: 36 (100%)<br>F: 0 (0%)<br>Setting:<br>Institution	NA<br>Diagnosis tool:<br>NA<br>Severity:<br>moderate to severe	Total sessions: NI<br>Total week: 36<br>Format: group<br>Control:<br>Usual care		
32.	Serrano et al.<br>(2004)<br>Spain	Sample size<br>Overall: 43<br>EG: 20<br>CG: 23<br>Mean (SD) age:<br>EG: 75.8±8.1<br>CG: 78.4±7.3<br>Gender:<br>M: 10 (23.3%)<br>F: 33 (76.7%)<br>Setting:<br>Community	Type:<br>Depression<br>Duration:<br>NA<br>Diagnosis tool:<br>NA<br>Severity:<br>Mild to severe	Experiment:<br>Life review therapy<br>Frequency: 1x/per week<br>Duration: NI<br>Total sessions: 4<br>Total week: 4<br>Format: individual<br>Control:<br>No intervention	Psychological:<br>Depression (CES-D)<br>Life satisfaction<br>(LSIA)	- Baseline<br>- Post-test (6 weeks)
33.	Stevens-<br>Ratchford<br>(1993)<br>USA	Sample size<br>Overall: 24<br>EG: 12<br>CG: 12<br>Mean (SD) age:<br>79.75 (NI)<br>Gender:<br>M: 8 (33.3%)<br>F: 16 (66.7%)<br>Setting:<br>Institution	Type:<br>No depression<br>Duration:<br>NA<br>Diagnosis tool:<br>NA<br>Severity:<br>NA	Experiment:<br>Life review<br>Frequency: 2x/per week<br>Duration: 120 mins<br>Total sessions: 6<br>Total week: 3<br>Format: group<br>Control:<br>Usual care	Psychological:<br>Self-esteem (RSES)<br>Depression (BDI)	- Baseline<br>- Post-test (4 weeks)
34.	Subramaniam<br>et al.<br>(2014)<br>UK	Sample size<br>Overall: 23<br>EG: 11<br>CG: 12<br>Mean (SD) age:<br>EG: 84.5±6.7<br>CG: 88.3±6.0<br>Gender:<br>M: 7 (30.4%)<br>F: 16 (69.6%)<br>Setting:	Type:<br>Dementia<br>Type of dementia:<br>NA<br>Duration:<br>NA<br>Diagnosis tool:<br>DSM-III<br>CDR<br>Severity:<br>Mild to moderate	Experiment:<br>Life review<br>Frequency: 1x/per week<br>Duration: 30-60 mins<br>Total sessions: 12<br>Total week: 12<br>Format: individual<br>Control:<br>Usual care	Psychological:<br>Depression (GDS)	- Baseline<br>- Post-test (12 weeks)<br>- Follow-up (6 weeks)

		Institution				
35.	Van Bogaert et al. (2016) Belgium	Sample size<br>Overall: 60<br>EG: 29<br>CG: 31<br>Median age (IQR):<br>EG: 84 (79.5-90.5)<br>CG: 84 (76-89)<br>Gender:<br>M: 12 (20%)<br>F: 48 (80%)<br>Setting:<br>Institution	Type:<br>Neurocognitive disorder<br>Type of Neurocognitive disorder:<br>NA<br>Duration:<br>NA<br>Diagnosis tool:<br>DSM-V<br>MMSE<br>Severity:<br>Mild to moderate	Experiment:<br>Reminiscence intervention<br>Frequency: 2x/per week<br>Duration: 45 mins<br>Total sessions: 16<br>Total week: 8<br>Format: individual<br>Control:<br>Normal care	Psychological:<br>Depression (CSDD)<br>Global cognitive function:<br>Cognitive function (MMSE)	- Baseline<br>- Post-test (8 weeks)
36.	Wang (2007) Taiwan	Sample size<br>Overall: 102<br>EG: 51<br>CG: 51<br>Mean (SD) age:<br>EG: 79.8±6.3<br>CG: 78.9±7.6<br>Gender:<br>M: 50 (49%)<br>F: 52 (51%)<br>Setting:<br>Community	Type:<br>Dementia<br>Type of dementia:<br>NA<br>Duration:<br>EG: 1.8±1.2<br>CG: 2.7±3.0<br>Diagnosis tool:<br>CDR<br>Severity:<br>Mild to moderate	Experiment:<br>Reminiscence therapy<br>Frequency: 1x/per week<br>Duration: 60 mins<br>Total sessions: 8<br>Total week: 8<br>Format: group<br>Control:<br>Usual care	Psychological:<br>Depression (CSDD)<br>Global cognitive function:<br>Cognitive function (MMSE)	- Baseline<br>- Post-test (8 weeks)
37.	Wu & Malcolm (2016) Taiwan	Sample size<br>Overall: 103<br>EG: 53<br>CG: 50<br>Mean (SD) age:<br>EG: 73.5±7.3<br>CG: 73.6±7.6<br>Gender:<br>M: 32 (31.1%)<br>F: 71 (68.9%)<br>Setting:<br>Community	Type:<br>Dementia<br>Type of dementia:<br>AD: 102 (99%)<br>Frontotemporal dementia: 1 (1%)<br>Duration:<br>NA<br>Diagnosis tool:<br>MMSE<br>Severity:<br>Mild to moderate	Experiment:<br>Spiritual reminiscence intervention<br>Frequency: 1x/per week<br>Duration: 60 mins<br>Total sessions: 6<br>Total week: 6<br>Format: group<br>Control:<br>Usual care	Psychological:<br>Life satisfaction (LSS)<br>Global cognitive function:<br>Cognitive function (MMSE)	- Baseline<br>- Post-test (6 weeks)

Abbreviations: AD: Alzheimer's Disease; ADAS-cog: Alzheimer's Disease Assessment Scale - cognitive subscale; ADRDS: Alzheimer's Disease and Related Disorders Association Scale; BDI: Beck Depression Inventory; BSI-18: Brief Symptom Inventory-18; CDR: Clinical Dementia Rating; CES-D: Center for Epidemiologic Studies Depression Scale; CSDD: Cornell Scale for

Depression in Dementia; DSM-III: Diagnostic and Statistical Manual of Mental Disorders-III; DSM-IV: Diagnostic and Statistical Manual of Mental Disorders-IV; DSM-V: Diagnostic and Statistical Manual of Mental Disorders-V; EDI: Elderly Depression Inventory; EQ-5D: EuroQol 5-Dimension; GDS: Global Deterioration Scale; GDS-15: Geriatric Depression Scale-15 item; LBD: Lwey Body Disease; LSI-A: Life Satisfaction Index–Aged; LSS-C: Life Satisfaction Scale-Chinses; MMSE: Mini-Mental State Examination; MSQ: Mental Status Questionnaire; NA: Not Available; NI: No Information; NINDS: National Institute of Neurological and Communicative Disorders and Stroke; PD: Parkinson's Disease; RSES: Rosenberg Self-Esteem Scale; RULS-V3: Revised University of California Los Angeles Loneliness Scale; SLS: Satisfaction with Life Scale; UCLA: University of California, Los Angeles Loneliness Scale; VD: Vascular Dementia

Figure S1. Risk of bias assessment.

Study ID	D1	D2	D3	D4	D5	Overall
Alfonso & Bueno, 2009	!	+	+	+	+	!
Aydogdu et al., 2022	+	+	+	+	+	+
Balci et al. 2022	!	+	+	+	+	!
Brazrafshan et al., 2022a	+	+	+	+	+	+
Bazrasfshan et al., 2022b	!	+	+	+	+	!
Chan et al., 2013	!	+	+	+	+	!
Chiang et al., 2010	!	+	+	+	+	!
Chippendale & Bear-Leahman, 2012	!	+	+	+	+	!
Choy & Lou, 2016	!	+	+	+	+	!
Cook 1991	!	+	+	+	!	!
Deponte & Missan, 2007	!	+	+	+	!	!
Duru Asiret & Dutkun, 2018	+	+	+	+	+	+
Goncalves et al., 2009	!	!	+	+	+	!
Hojjati	+	+	+	+	+	+
Hsieh	!	+	+	+	+	!
Ilali	!	+	+	+	+	!
Jahanbin	!	+	+	+	+	!
Justo-Henriques (a)	+	+	+	+	+	+
Justo-Henriques (b)	+	+	+	+	+	+
Keisari	!	+	+	+	+	!
Lan	+	+	+	+	!	!
Lan (b)	!	+	+	+	!	!
Li, 2020	+	+	+	+	!	!
Li (2022)	!	+	+	+	!	!
Mastel-Smith	!	+	+	+	!	!
Musavi	-	+	+	+	!	-
Nomura	!	+	+	+	!	!
Perez-Saez 2022	+	+	+	+	!	!
Pishvaei 2015	!	+	+	+	!	!
Preschl 2012	!	+	+	+	!	!
Robb et al. 1986	-	!	+	+	+	-
Serrano 2004	!	+	+	+	!	!
Stevens-Ratchford 1993	!	+	+	+	!	!
Subramaniam 2014	!	+	+	+	!	!
Van Bogaert, 2016	!	+	+	+	+	!
Wang 2007	!	+	+	+	!	!
Wu 2016	+	+	+	+	+	+

+ Low risk
! Some concerns
- High risk

D1 Randomisation process
D2 Deviations from the intended interventions
D3 Missing outcome data
D4 Measurement of the outcome
D5 Selection of the reported result

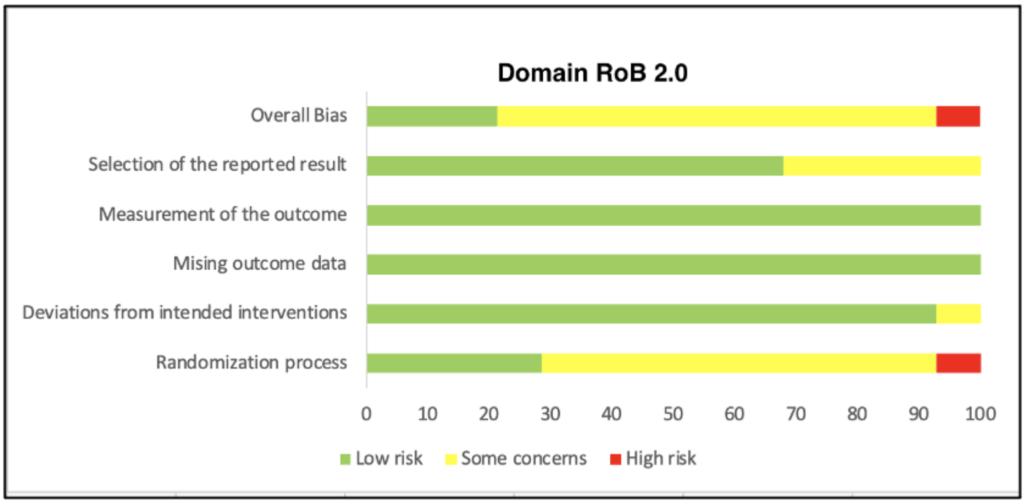

Table S5. Sensitivity analyses.

Outcomes	Number of studies	Sensitivity	I^2	Hedge's g	95% Confidence interval	*P-value*
Depression						
Post-intervention effects	26	Remove one study	84.905%	-0.821	-1.080, -0.562	<0.001
		Remove one high risk study	84.976%	-0.807	-1.073, -0.541	<0.001
Follow-up effects	5	Remove one study	94.909%	-1.397	-2.452, -0.341	0.009
Self-esteem						
Post-intervention effects	7	Remove one study	87.800%	0.977	0.312, 1.642	0.004
Life satisfaction						
Post-intervention effects	13	Remove one study	63.829%	0.626	0.373, 0.878	<0.001
Loneliness						
Post-intervention effects	4	Remove one study	97.080%	-2.221	-3.798, -0.643	0.006
Follow-up effects	3	Remove one study	97.983%	-3.483	-6.023, -0.943	0.007
Global cognitive function						
Post-intervention effects	12	Remove one study	25.207%	0.342	0.193, 0.491	<0.001
		Remove one high risk study	18.082%	0.363	0.221,0.504	<0.001
Follow-up effects	4	Remove one study	87.800%	-0.320	-0.908, 0.268	0.286

Figure S2. Funnel plot on depression.

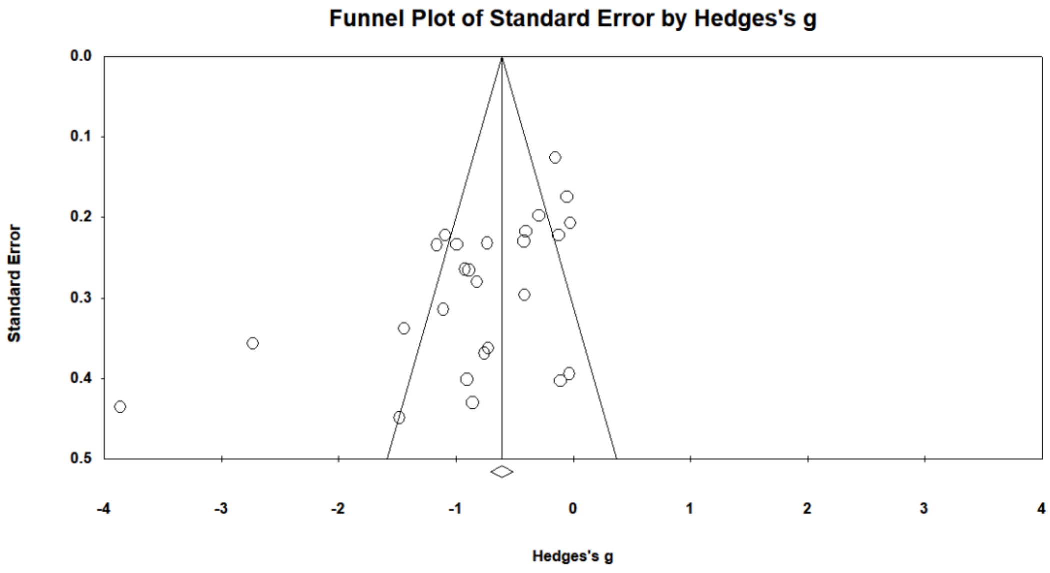

Figure S3. Funnel plot on life satisfaction.

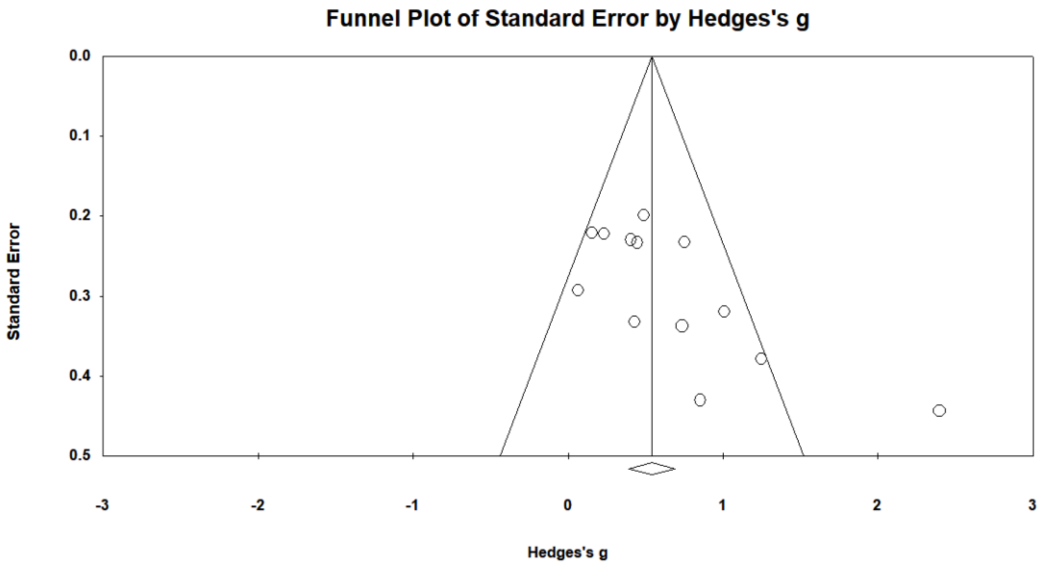

Figure S4. Funnel plot on global cognitive function.

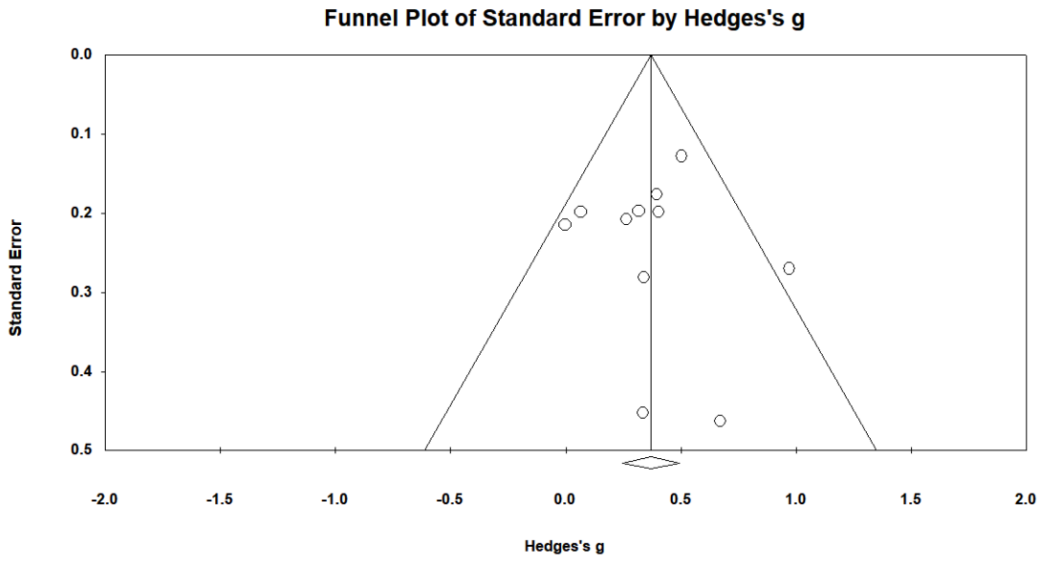

Table S6. Publication bias.

Outcomes	Number of studies	Studies trimmed	Observed value		Adjusted values		Egger's test
			Hedges' g	95% Confidence interval	Hedges' g	95% Confidence interval	*P-value*
Depression							
Post-intervention effects	26	8	-0.821	-1.080, -0.562	-1.154	-1.464, -0.844	0.001
Follow-up effects	5	0	-1.397	-2.452, -0.341			0.248
Self-esteem							
Post-intervention effects	7	0	0.977	0.312, 1.642			0.128
Life satisfaction							
Post-intervention effects	13	4	0.626	0.373, 0.878	0.407	0.119, 0.694	0.011
Loneliness							
Post-intervention effects	4	0	-2.221	-3.780, -0.643			0.057
Follow-up effects	3	0	-3.483	-6.023, -0.943			0.183
Global Cognitive function							
Post-intervention effects	12	0	0.342	0.193, 0.491			0.635
Follow-up effects	4	0					0.011
